# Chiral Dysprosium-[7]Helicene Macrocycles Showing Record Single-Molecule Magnet Properties in the Lanthanide–Helicene Family

**DOI:** 10.1021/jacs.5c15088

**Published:** 2025-11-10

**Authors:** Zhenhua Zhu, Tingting Wang, Lorenzo A. Mariano, Sagar Paul, Wolfgang Wernsdorfer, Alessandro Lunghi, Jinkui Tang

**Affiliations:** † State Key Laboratory of Rare Earth Resource Utilization, Changchun Institute of Applied Chemistry, 58277 Chinese Academy of Sciences, Changchun 130022, P. R. China; ‡ School of Applied Chemistry and Engineering, University of Science and Technology of China, Hefei 230026, P. R. China; § School of Physics, AMBER and CRANN Institute, 8809 Trinity College, Dublin 2, Ireland; ∥ Physikalisches Institut, Karlsruhe Institute of Technology (KIT), Karlsruhe D-76131, Germany; ⊥ Institute for Quantum Materials and Technology (IQMT), Karlsruhe Institute of Technology (KIT), Eggenstein-Leopoldshafen, Karlsruhe D-76344, Germany

## Abstract

Chiral helicene-based metal complexes have emerged as an extremely promising class of multifunctional molecules for a wide range of applications. Despite significant progress in the synthesis of helicene-based transition-metal complexes in recent decades, lanthanide species lag far behind and their number is very limited. Compared to the widely studied optical activity and magneto-chiroptical effects, the single-molecule magnet (SMM) properties of lanthanide–helicene compounds, in particular their spin relaxation dynamics, are still largely underexplored. In this work, starting from the chiral Dy­(III) hexaazamacrocycles, we prepared the first lanthanide–[7]­helicene enantiomers using [7]­helicene-2-hydroxy as an axial ligand. The introduction of [7]­helicene not only endows the compounds of relatively large molar absorptivities but also land them extremely high thermal stability with decomposition temperature beyond 320 °C. Remarkably, in terms of the traditional metrics of SMMs, these molecules are the best lanthanide–helicene SMMs to date, showing an effective energy barrier (*U*_eff_) exceeding 600 cm^–1^ and open hysteresis loops at zero field up to 12 K using a sweep rate of 200 Oe/s. Ab initio spin dynamics calculations and phonon analysis reveal that vibrations of the equatorial benzene rings that are in *trans*-diaxial manner and the two nearest nitrogen atoms dominantly contribute to the Orbach relaxation, driving the relaxation of the magnetization via the third Kramers doublet. Raman relaxation, responsible for the hysteresis closure at 12 K, is instead driven by delocalized phonons involving rigid movements of the ligands, counterions, and solvent. These findings pave a new way toward preparing and modulating magnetodynamics of lanthanide–helicene compounds.

## Graphical abstract

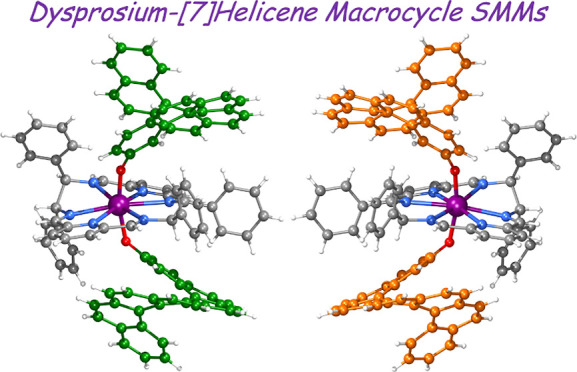

## Introduction

Helicenes, featuring intrinsic helical chirality due to the nonplanar screw-shaped skeletons in spite of the absence of any stereogenic center (see Figure 1A),^1^ have attracted great attention in the past few decades not only as they exhibit chirality-related phenomena but also they possess abundant electric, optical, and magnetic properties,^2^ which enable their use for a wide range of applications in asymmetric catalyst,^3^ organic semiconductors,^4^ spintronics,^5^ etc. The pioneer work of the first synthesis and resolution of carbo[6]­helicene reported by Newman and Lednicer has given helicene chemistry a vibrant life.^6^ Since then, rapid progress has been witnessed in preparing carbohelicenes with an increasing number of fused rings, heterohelicenes containing various main-group elements and multiple helicenes with appealing topological structures and chiroptical properties.^2,7^ Moreover, organic chemists have developed numerous innovative protocols to prepare enantiopure helicenes and introduce various functional groups onto the helicene scaffold, e.g., transition-metal-catalyzed [2 + 2 + 2] cycloadditions,^8^ Rh-/Pd-catalyzed enantioselective C–H activation,^9,10^ and visible-light-mediated photoredox catalysis,^11^ which renders helicenes as versatile chiral­(pro)­ligands to construct chiral metal complexes and their supramolecular assemblies. Thanks to the general toolbox provided by coordination and organometallic chemistry, transition-metal–helicene compounds have so far covered 12 kinds of cations such as Ag­(I),^12^ Cu­(I),^13^ Zn­(II),^14^ and Pt­(II)^15^ and involved diverse coordination assemblies, e.g., helical conjugated ladder polymers,^16^ supramolecular cage,^17^ and helicenic metallocenes.^18^ However, compared to transition-metal–helicene compounds, lanthanide-based ones are still in their infancy,^19,20^ and the numbers reported to date have been fairly limited (Figure 1B).

**Figure 1:**
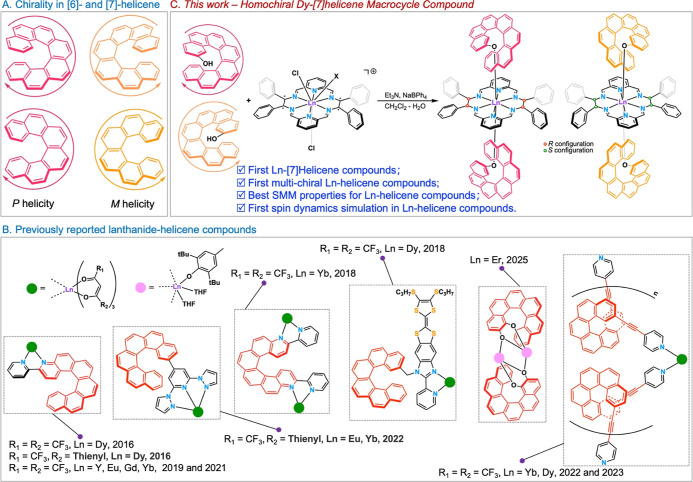
(A) Molecular structures of [*n*]­helicene (*n* = 6 and 7) enantiomers; (B) lanthanide–helicene family with determined solid structures; (C) synthetic routes of **1** and **2**.

The first lanthanide–helicene compounds, [Dy­(hfac)_3_(L_1_)] (hfac = hexafluoroacetylacetonate, L_1_ = 3-(2-pyridyl)-4-aza[6]-helicene), whose racemic and optically pure forms are both zero-field single-molecule magnets (SMMs), were synthesized by Ou-Yang et al. in 2016.^21^ During the following ten years, six kinds of [6]­helicene-based ligands were designed to be combined with various lanthanide ions with different types of magnetic anisotropy and electron energy levels, showing promising potential in magneto–optical cross effects and multifunctional lanthanide materials. In 2021, Atzori and co-workers pioneered the detection of strong magneto-chiral dichroism (MChD) signals in the near-infrared (NIR) spectral range with the help of a pair of enantiomers of Yb­(III)–[6]­helicene compounds that are isostructural to the above Dy­(III) species.^22^ In the following year, Dhbaibi et al. decorated the [6]­helicene with two pyridine coordinating groups and employed it to react with Yb­(hfac)_3_·2H_2_O, obtaining the first homochiral 1D lanthanide–helicene coordination polymer (the first one on the right in Figure 1B).^23^ The latter compound displays the coexistence of field-induced SMM behaviors, NIR-circularly polarized luminescence (CPL), and room-temperature MChD. However, there was hardly any progress in enhancing their SMM properties during this period primarily due to two fundamental challenges: (i) in all reported lanthanide–helicene compounds, most of helicene units are derivatives of pyridine or 2,2′-bipyridine, serving as the auxiliary ligand; the crystal-field splitting is largely governed by the interaction, both electrostatic and covalent in nature,^24,25^ between lanthanide ions and the enolate anion of β-diketone in lanthanide precursors, and (ii) the extremely limited choice of the helicene scaffolds due to their very intricate and laborious synthesis and chiral separation. As such, the promising milestone of constructing high-performance lanthanide–helicene SMMs is yet to be achieved.

In recent years, lanthanide macrocycle compounds have sparked enormous attention in multidisciplinary field owing to their wide applications, such as magnetic resonance imagining (MRI),^26,27^ molecular theranostics,^28^ CPL,^29^ biomolecule detection,^30^ and molecular magnetism.^31^ In particular, chiral lanthanide hexaazamacrocycles (Figure 1C) built from [2 + 2] imine condensations between chiral vicinal diamines^32^ and pyridine-2,6-dicarbaldehyde or its derivates^33^ with lanthanide ions as templates have exhibited easy modification in both equatorial plane and axial positions,^34,35^ offering diverse possibilities to integrate with helicene ligands. Furthermore, such hexaazamacrocycles possess large internal cavities capable of encapsulating lanthanide ions within a planar structure, creating a high local symmetry and relatively weak transverse crystal field (CF) for ions with prolate ground-state 4f electron density, e.g., Dy­(III). Herein, we report the first pair of enantiomers of the Dy­(III)–[7]­helicene compound (Figure 1C), where the helicene scaffold is decorated with the hydroxy group to induce strong axial CF by virtue of the strong interaction between phenolate and Dy­(III) ion.^36,37^ They showed the effective energy barrier to magnetization reversal (*U*_eff_) exceeding 600 cm^–1^ and open hysteresis loops at zero field up to 12 K. While these two metrics would not be considered competitive with the best-performing examples,^38^ they far surpass all previously reported lanthanide–helicene SMMs. Complementing these findings, ab initio spin dynamics calculations and phonon analysis were carried out, elucidating the magnetic relaxation mechanism for both enantiomers. It is shown that the strongest coupled phonons arise from the vibrations of the equatorial benzene rings and the two nearest nitrogen atoms, contributing to the high-temperature Orbach relaxation, while delocalized soft vibrational modes drive low-temperature Raman relaxation.

## Results and Discussion

### Design, Synthesis, Structure, and Optical Properties of Dy­(III)–[7]­Helicene

As shown in Scheme S1, racemic [7]­helicene-2-hydroxy (HL) was synthesized by a nine-step process from two commercially cheap reactants, benzaldehyde and diethyl (4-bromobenzyl)­phosphonate, involving three times of Horner–Wadsworth–Emmons (HWE) reaction^39^ (Figures S1–S9). Then, it was used directly without chiral resolution to replace Cl^–^ in the Dy­(III) macrocycle precursor, Dy­(L^N6^)­Cl_2_X (L^N6^ is homochiral hexaazamacrocycles deriving from [2 + 2]-condensation between optically pure 1,2-diphenylethylenediamine and pyridine-2,6-dicarbaldehyde with Dy­(III) ion as a template, X = H_2_O or CH_3_OH), leading to a pair of Dy­(III)–[7]­helicene enantiomers formulated as [Dy­(L^N6^)­(L)_2_]­[BPh_4_]·3CH_2_Cl_2_, **1** (L^N6^ = *RRRR*-isomer of the macrocycle, L = deprotonated [7]­helicene-2-hydroxy possessing *P* helicity) and **2** (L^N6^ = *SSSS*-isomer of the macrocycle and L = deprotonated [7]­helicene-2-hydroxy possessing M helicity) (Figures 1C and S10). This means that the homochiral Dy­(III) macrocycle precursor could resolve racemic [7]­helicene-2-hydroxy. The mechanism of chiral recognition and enantioselective separation of chiral lanthanide hexaazamacrocycle for hydroxy-modified [*n*]­helicene, *n* = 5, 6, 7, will be elucidated in a forthcoming study. The complexes are highly thermally stable with the decomposition temperature up to 325 °C (Figure S11), which is even larger than that of fluorinated Dy­(III) macrocycles (280 °C).^34^ Red rod-shaped crystals of **1**/**2** could be obtained by slow diffusion of pentane into a saturated solution of CH_2_Cl_2_ at room temperature. Their solid structures, determined by single-crystal X-ray diffraction (SC-XRD), are shown in Figure 2. They both crystallized in the chiral polar space group, *P*2_1_ (Table S1), exhibiting distorted hexagonal bipyramid local coordination geometry (Table S4). Considering their enantiomeric relationship, to avoid redundancy, here, we only describe the structural features of **1**. However, it should be noted that certain structural and magnetic property differences still exist between **1** and **2** (vide infra), which may be related to the differing positions of solvent molecules within the crystal lattice. As shown in Table S2, the Dy–N bonds in the equatorial plane range from 2.608(9) to 2.683(8) Å, averaged to 2.65 Å, presenting close similarities to other quasi-*D*_6*h*_ lanthanide hexaazamacrocycles.^31,40,41^ It is worth noting that two pairs of phenyl substituents in two chiral diamine units displayed completely different conformations (Figure 2A), one is in *trans*-diequatorial manner and the other is in *trans*-diaxial manner. To the best of our knowledge, this is the first example of observing diaxial conformation of *trans*-disubstituted diamines in lanthanide macrocyclic and salen complexes,^42,43^ which is due to the unique π-conjugated screw-shaped structure of [7]­helicene at the axial positions. The axial Dy–O bond lengths, 2.106(8) and 2.131(8) Å, are much shorter than those of Dy–N, owing to much stronger 4f-phenolate interaction. The O–Dy–O angle at 165.8(3)° is smaller than those induced by the Ph_3_SiO^–^ anion (Table S3),^31,34^ suggesting a larger steric repulsion between the [7]­helicene ligand and equatorial macrocycle plane. In turn, the Dy­(III) macrocycle plane also modulates the distortion of the [7]­helicene skeleton, as quantified by torsional angles and interplanar angles (Figure 2B, Tables S5 and S25 –S28). Compared to the free ligand, HL, the sum of the internal three dihedral angles of the inner rim in **1** remains almost unchanged, 76.645° vs 77.737°, while the outer dihedral angle C_1_–C_2_–C_3_–C_4_ increased from 10.583° to 12.919° and the other one C_5_–C_6_–C_7_–C_8_ decreased from 15.264° to 13.612°. The interplanar angles between the two terminal benzene rings for HL and **1** are, respectively, 46.311° and 45.448°, and both are much larger than that in [7]­helicene (32.388°).

**Figure 2:**
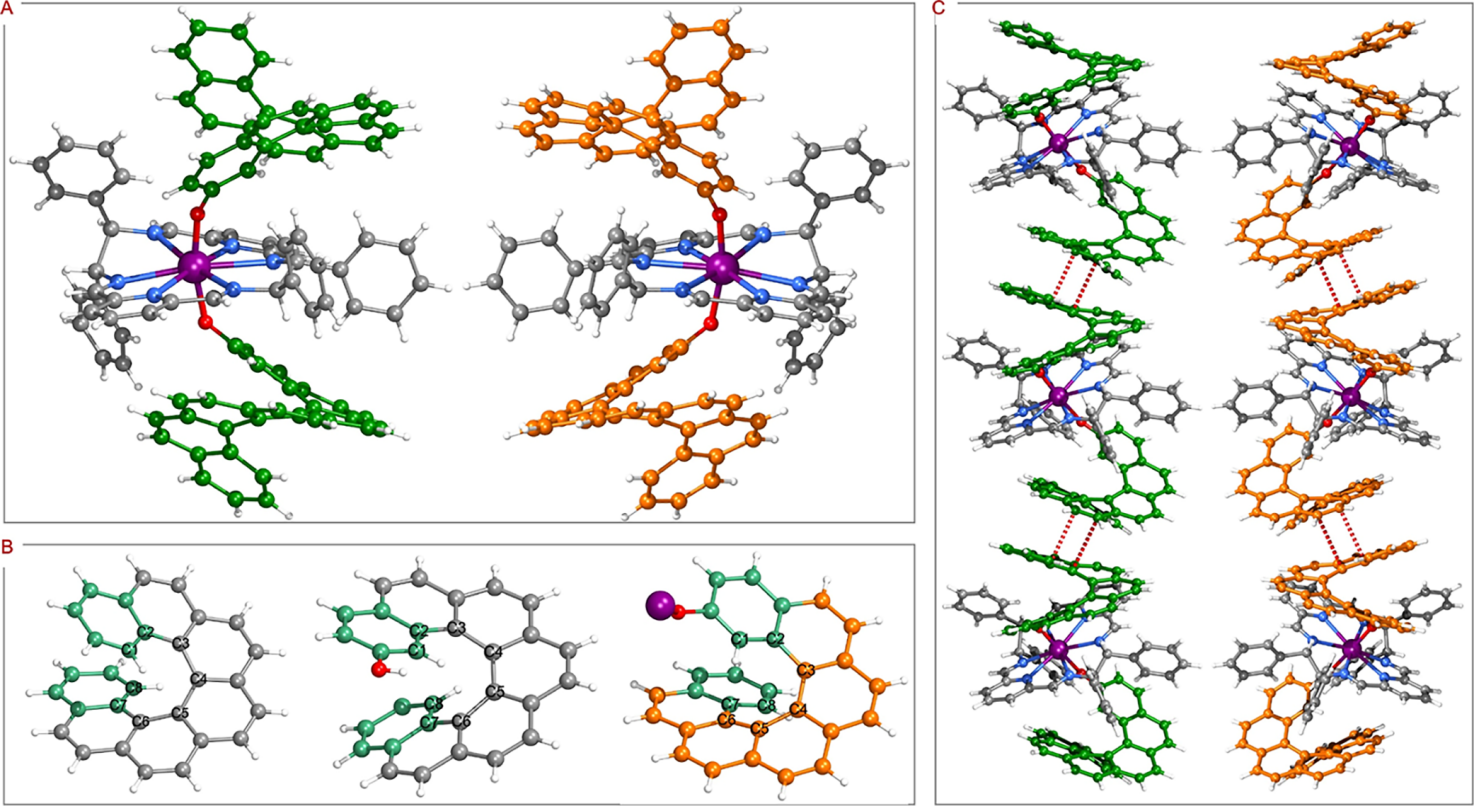
(A) Solid structures of **1** (left) and **2** (right). Noncoordinated BPh_4_^–^ and CH_2_Cl_2_ molecules are omitted for clarity. (B) Torsional angle, from C1 to C8, and interplanar angle between two sea-green terminal benzene rings in [7]­helicene (left),^44^ HL (middle) and *P*-L in **1** (right). See Table S5 for details. (C) Crystal packing of **1** (left) and **2** (right), along the *c* axis. Dy, purple; C, gray/green/orange/sea-green; N, blue; O, red; H, white.

The crystal packings along crystallographic *c* axis of **1** and **2** are shown in Figure 2C. The one-dimensional columnar arrangement is observed in the homochiral crystal via the intermolecular π···π interactions with face-to-face contact between the center of the benzene rings of 3.80 Å and the angle between the planes of benzene rings of 17.2°. The shortest intermolecular Dy···Dy distance was also determined to be 13.258(8) and 13.287(7) Å for **1** and **2**, respectively, by means of crystal packing analysis, implying negligible intermolecular magnetic interactions (Figures S29 and S30).

Figure 3 compares the UV–vis absorption spectra of HL and **1** in CH_2_Cl_2_, showing the absorption edges at 418 and 435 nm, respectively. The two most intense absorption peaks for them are both at 233 and 268 nm, respectively; however, the compound exhibits much larger molar absorptivities up to 128,000 and 152,000 M^–1^ cm^–1^, respectively. Notably, the other three minor absorption bands for **1** around 304, 328, and 368 nm also show a high molar absorption coefficient of 50,600, 38,300, and 17,000 M^–1^ cm^–1^, respectively, reflecting the relatively strong light-absorbing ability of [7]­helicene-2-hydroxy (Figures S12–S24). The circular dichroism (CD) spectra of *P*-HL/**1** and *M*-HL/**2** displayed analogous patterns. As expected, the spectra for **1** and **2** are perfectly mirror-symmetric showing two strong Cotton effects at around 270 and 356 nm, respectively, with opposite sign, confirming their enantiomeric relationship.

**Figure 3:**
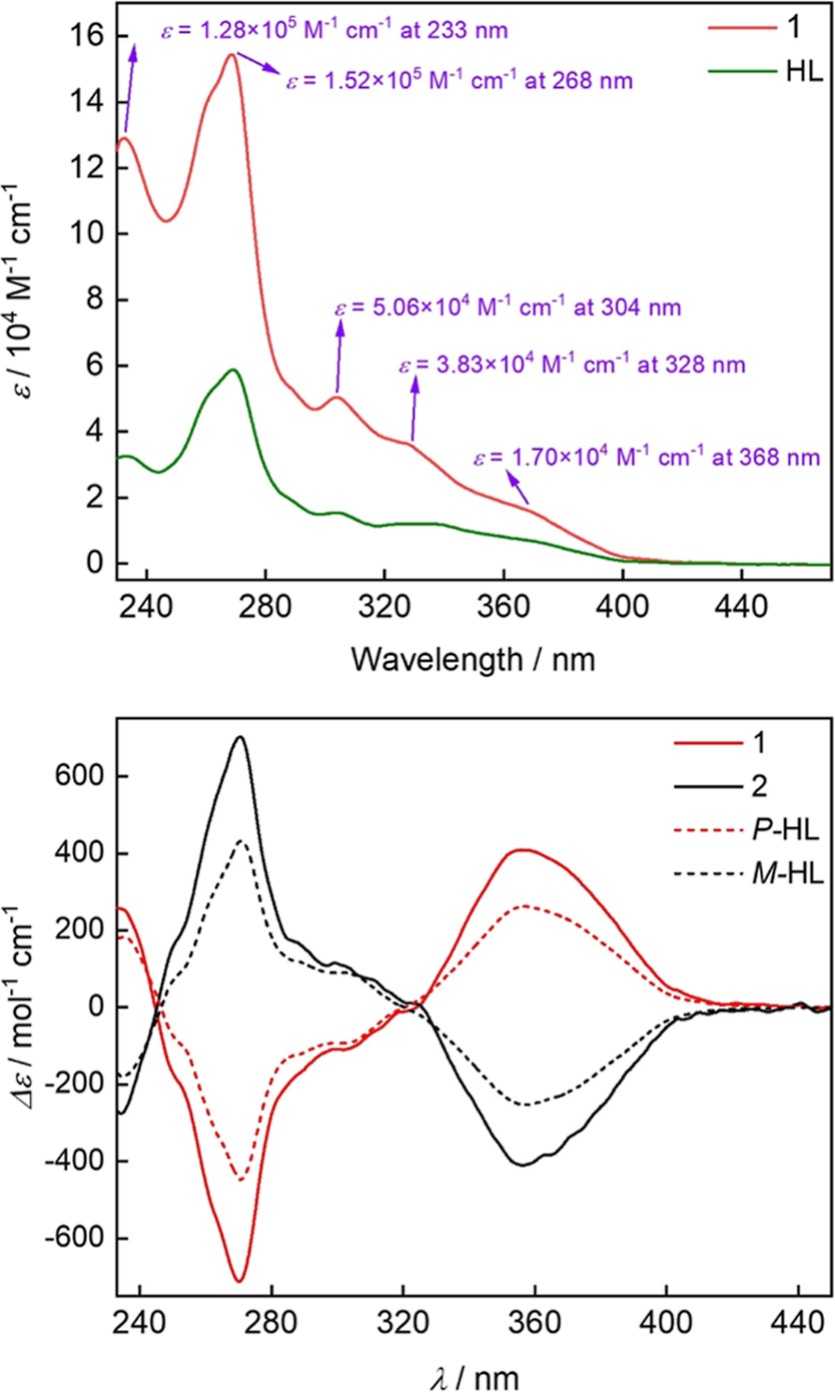
(Top) UV–vis absorption of **1** and HL in CH_2_Cl_2_ (*c* = 7.5 × 10^–6^ M) and (bottom) CD spectra of two pairs of enantiomers in CH_2_Cl_2_, *P*-/*M*-HL (*c* = 1.0 × 10^–5^ M) and **1**/**2** (*c* = 1.0 × 10^–6^ M).

### Magnetic Properties

The static and dynamic magnetic properties of these two compounds were thoroughly investigated in experiment using both polycrystalline samples and single crystals via a superconducting quantum interference device (SQUID) magnetometer and μ-SQUID technologies. The χ_M_ *T* vs *T* (χ_M_ is molar magnetic susceptibility) profiles (Figures S31 and S32), measured at a direct current (dc) magnetic field of 1000 Oe and in the temperature of 2.0–300 K, give the values at 300 K of 13.92 and 13.97 cm^3^ K mol^–1^ for **1** and **2**, respectively, close to the theoretical value of 14.17 cm^3^ K mol^–1^ for a free Dy­(III) ion with a ^6^H_15/2_ ground state. Upon cooling, the χ_M_ *T* values first exhibited gradual decline until ca. 7 K, followed by a sudden drop yielding values of 9.11 and 8.65 cm^3^ K mol^–1^ at 2.0 K, which are attributed to the thermal depopulation of excited *m*_J_ states of Dy­(III) ions and magnetic blocking, respectively. Isothermal field-dependent magnetization experiments were performed at 1.9, 3.0, and 5.0 K from 0 to 7 T (Figures S33 and S34). The *M* vs *H* curves showed that (i) pronounced S-shape at 1.9 K and low fields, reconfirming the magnetic blocking at low temperatures,^45–47^ and (ii) the magnetization at 1.9 K, respectively, saturates at 5.26 and 5.35 μ_B_ under a 7 T dc field for **1** and **2**. Both values are approaching 5.00 μ_B_, implying the strong magnetic anisotropy (*m*_J_ = ± 15/2 ground states, *g*_eff,z_ = 20) in the investigated systems. To verify their magnetic memory effect, *M*(*H*) hysteresis loops were examined between ±7 T using a sweep rate of 200 Oe/s^–1^. As illustrated in Figures 4 and S35 –S37, the present system exhibits open loops at zero field up to at least 12 K. The abrupt drop of the magnetization around zero field indicates the presence of quantum tunneling of the magnetization (QTM) process, which was also revealed by μ-SQUID and alternate-current (ac) susceptibility measurements. The single-crystal *M*(*H*) loops at sub-Kelvin temperatures (Figure 4C,D) clearly exhibit a fast QTM process at zero field and slow magnetic relaxation at low fields.

**Figure 4:**
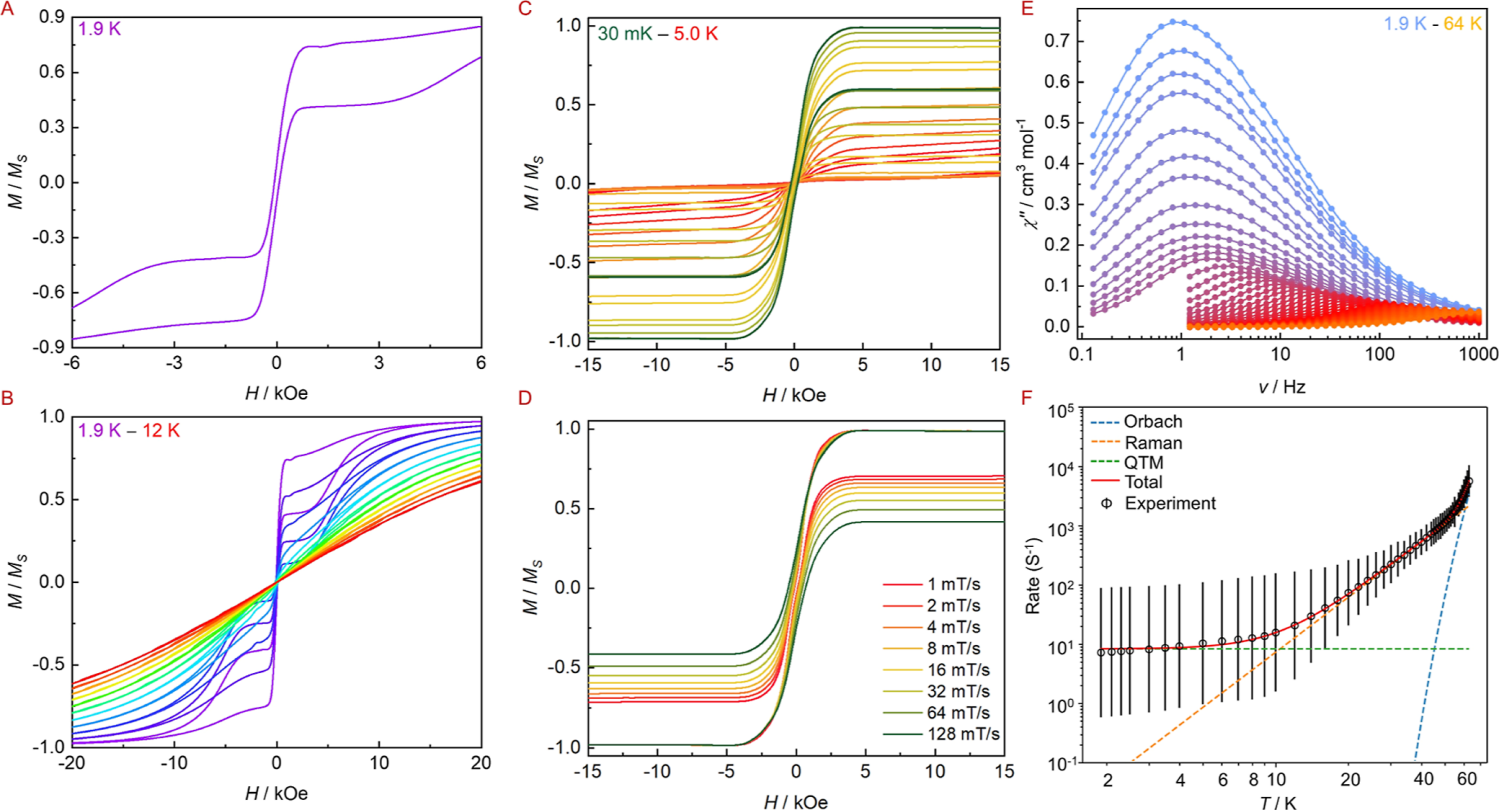
Magnetic properties for **1**. (A,B) Normalized *M*(*H*) loops at a fixed temperature *T* = 1.9 K and different temperatures (1.9–12 K) with a field sweep rate of 200 Oe/s; (C,D) single-crystal magnetic hysteresis loops measured by μ-SQUID in the temperature of 30 mK–5 K at a field sweep rate of 16 mT/s and different field sweep rates at a fixed temperature of 30 mK; (E) χ″(ν) plots at a zero applied dc field in the temperature of 1.9 to 64 K; (F) temperature dependence of the relaxation time τ. The black circles and red solid line represent experimental data and fit, respectively. Solid black lines indicate error bars from the distributions of relaxation times.

To assess the magnetization dynamics and gain insights into the relaxation mechanisms of the magnetization in this system, we carried out ac susceptibility measurements for **1** and **2** using an oscillating ac magnetic field of 3 Oe in the frequency range of 0.1 to 1000 Hz (Figures 4E and S38 –S39). These two exhibited similar and detectable out-of-phase signals (χ″) under a zero dc field, which exhibit well-defined peaks between 5 and 64 K, unambiguously verifying their SMM behaviors. At temperatures above 5 K, the intensities and the positions of peaks in χ″(ν) plots decreased and shifted to higher frequencies, respectively, with the temperature increasing, while at a low-temperature region, the positions remain almost unchanged indicative of a temperature-independent QTM process. The magnetic relaxation times (τ) at each measurement temperature were extracted by fitting Cole–Cole plots to the generalized Debye model (Figures S41–42 and Tables S6–S7) and plotted as τ^–1^ vs *T* (Figures 4F and S43). The temperature at which the Raman relaxation mechanism becomes dominant over the Orbach process was determined by the maximum in the second derivative of the log­(1/τ) vs log­(*T*) plot (Figures S40 and S43).^48^ In the temperature range of 54–64 K, the plot is linear corresponding to the thermally activated Orbach relaxation process. For the temperatures between 5 and 54 K (middle temperature range), it becomes obviously curved implying that the Raman relaxation process dominates. When the temperatures are below 5 K, relatively fast relaxation of the magnetization took place via QTM effect. Consequently, the full magnetic relaxation should be divided into three processes and the equation τ^–1^ = τ_0_^–1^exp­(−*U*_eff_/*k*_B_ *T*) + *CT* ^*n*^ + τ_QTM_^–1^ was used to determine respective contributions, giving the fitting results: *U*_eff_ = 618(14) cm^–1^, τ_0_ = 1.2(4) × 10^–10^ s, *C* = 1.2(2) × 10^–2^ s^–1^ K^–*n*^, *n* = 2.90(6), τ_QTM_ = 0.13(1) s with adjusted *R* ^2^ = 0.997 for **1** and *U*_eff_ = 615(12) cm^–1^, τ_0_ = 1.4(4) × 10^–10^ s, *C* = 1.2(2) × 10^–2^ s^–1^ K^–*n*^, *n* = 2.90(5), τ_QTM_ = 0.14(1) s with adjusted *R* ^2^ = 0.998 for **2**. Although hysteresis temperature and *U*_eff_ value are much smaller than some [Dy­(Cp^R^)_2_]^+^ cations (where Cp^R^ is a substituted cyclopentadienyl anion) and the dysprosium bis­(amide)–alkene complex,^38,49–51^ they represent the best metrics for all reported helicene-based SMMs, confirming the sufficient uniaxial magnetic anisotropy of the ground state in **1** and **2**.

### Ab Initio Spin Dynamics Calculations and Phonon Analyses

In order to gain more insights on the magnetic relaxation mechanism of these compounds, we performed ab initio spin dynamics simulations. To accomplish this, we simulated from first-principles the phonons of the crystals’ unit cells, and their spin–phonon coupling. Starting from X-ray structures of **1** and **2**, cell and geometry optimization and simulations of Γ-point phonons have been performed with periodic density functional theory (pDFT) using the software CP2K.^52^ Cell optimization was performed employing a very tight force convergence criterion of 10^–7^ a.u. and SCF convergence criterion of 10^–10^ a.u. for the energy. A plane wave cutoff of 1000 Ry, DZVP-MOLOPT Gaussian basis sets, and Goedecker–Teter Hutter pseudopotentials^53^ were employed for all atoms. The Perdew–Burke–Ernzerhof (PBE)^54^ functional and DFT-D3 dispersion corrections^55^ were used. Phonon modes *Q*_α_ and frequencies ω_α_ were computed with a two-step numerical differentiation of forces and step 0.01 Å. Phonon spectra are reported in Figure S44.

Electronic structure and magnetic properties were computed on the pDFT-optimized structures using the software ORCA v. 5.^56^ The DKH-def2-TZVPP basis set was used for all atoms except Dy and H, for which SARC2-DKH-QZVP and DKH-def2-SVP were used, respectively. Douglas–Kroll–Hess (DKH) scalar relativistic correction to the electronic Hamiltonian was employed. Picture-change effects were included in the calculations. Multireference calculations were performed using Complete Active Space Self Consistent Field (CASSCF). The active space used to build the CASSCF wave function is (9,7), i.e., nine electrons in seven 4f orbitals. All possible states with multiplicity 6 were included in the state-average procedure. Mean-field spin–orbit coupling operator, along with Quasi Degenerate Perturbation Theory (QDPT), was employed to account for the mixing of spin-free states. The computed *g*-factors and Kramers pair energies are reported in Tables S10 and S11.

First- and second-order time-dependent perturbation theory have been used to simulate both one- and two-phonon relaxation processes. The software MolForge is used for these simulations.^57–60^ An effective crystal-field Hamiltonian of the form is used

1 $${Ĥ}_{CF}=\sum_{l=2,4,6}\sum_{m=-l}^{l}B_{m}^{l}{Ô}_{m}^{l}$$

where the operators ${Ô}_{m}^{l}$ are tesseral function of the total angular momentum operators, J⃗ . Once the eigenstates, |α⟩, and eigenvalues, *E*_α_, of these operators have been obtained, spin dynamics can be simulated by computing the transition rate among different spin states, *W*_ba_. Spin relaxation in molecular Kramers systems with large magnetic anisotropy takes contributions from one- and two-phonon processes. Considering one-phonon Orbach processes, the transition rate, *W*_ba_^1–ph^, among spin states reads

2 Ŵba1−ph=2πℏ2∑α|⟨b|(∂ĤCF∂Qα)|a⟩|G1−ph2(ωba,ωα)

where ℏω_ba_ = *E*_b_ – *E*_a_ and the term $\left(\partial {Ĥ}_{CF}/\partial Q_{\alpha }\right)$ provides the intensity of the coupling between spin and the α-phonon *Q*_α_. The function *G* ^1–ph^ reads

3 $$G^{1-ph}\left(\omega ,\omega_{\alpha }\right)=\delta \left(\omega -\omega_{\alpha }\right){\mathrm{n̅}}_{\alpha }+\delta \left(\omega +\omega_{\alpha }\right)\left({\mathrm{n̅}}_{\alpha }+1\right)$$

where ${\mathrm{n̅}}_{\alpha }={\left(e^{\hbar \omega_{\alpha }/k_{B}T}-1\right)}^{-1}$ is the Bose–Einstein distribution accounting for the phonons’ thermal population, *k*_B_ is the Boltzmann constant, and the Dirac delta functions enforce energy conservation during the absorption and emission of phonon by the spin system, respectively. Two-phonon Raman processes provide an alternative pathway of relaxation to equilibrium and the corresponding transitions *W*_ba_^2–ph^ can be expressed as

4 $${Ŵ}_{ba}^{2-ph}=\frac{2\pi }{\hbar^{2}}{\sum_{\alpha \beta }\left|T_{ba}^{\alpha \beta ,+}+T_{ba}^{\beta \alpha ,-}\right|}^{2}G^{2-ph}\left(\omega_{ba},\omega_{\alpha },\omega_{\beta }\right)$$

where the terms

5 $$T_{ba}^{\alpha \beta ,\pm }=\sum_{c}\frac{\left\langle b\left|\left(\partial H_{s}/\partial Q_{\alpha }\right)\right|c\right\rangle \left\langle c\left|\left(\partial H_{s}/\partial Q_{\beta }\right)\right|a\right\rangle }{E_{c}-E_{a}\pm \hbar \omega_{\beta }}$$

involve the contribution of all the spin states |*c*⟩ at the same time, often referred to as a virtual state. The function *G* ^2–ph^ fulfills a similar role as *G* ^1–ph^ for one-phonon processes and includes contributions from the Bose–Einstein distribution and imposes energy conservation. All the parameters appearing in eqs 2 and 4 are computed from first principles. The spin–phonon coupling coefficients $\left(\partial {Ĥ}_{CF}/\partial Q_{\alpha }\right)$ are obtained via two-step numerical differentiation of the crystal field parameters *B*_*m*_^*l*^ in eq 1 using a step of 0.01 Å. Once all the matrix elements *W*_ba_^*n*–ph^ have been computed, τ^–1^ can be obtained from the matrix elements of *W*_ba_^*n*–ph^. τ relaxation times due to Orbach and Raman mechanism are plotted in Figure 5A for both complexes against experimental results. Both compounds, **1** and **2**, exhibit similar relaxation mechanisms, and the observed differences in the computed τ values are attributed mostly to numerical precision in the geometrical optimization of the structures (Table S9). Therefore, for the sake of clarity, in the following, we report the analysis for **2**, while the corresponding analysis for **1** is provided in the Supporting Information (Figures S45–S48). The right panel of Figure 5 reports the transition rates between Kramers doublets. As expected, the data clearly shows that a direct transition within the ground-state KD is not possible due to the high axiality of Dy’s crystal field. However, several possible relaxation pathways are possible starting from the second excited KD.

**Figure 5:**
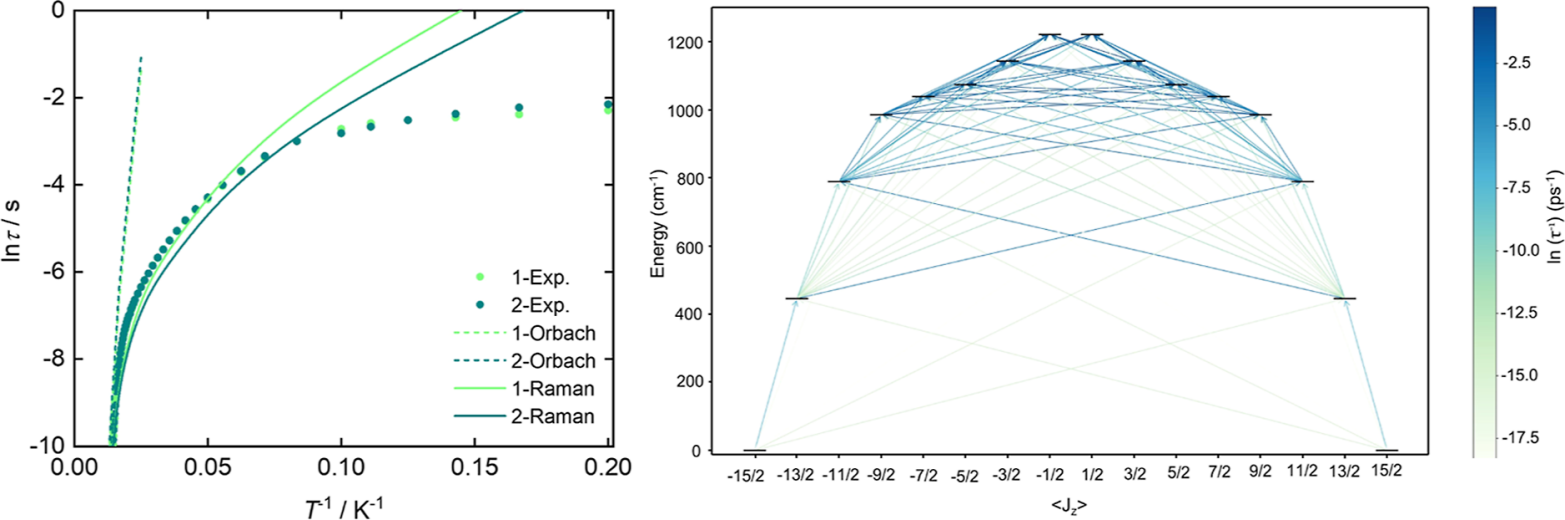
(Left) Magnetization reversal rates for **1** (green) and **2** (blue). The solid dots are the experimental rates, while the lines are the ab initio calculated Orbach (dashed) and Raman (solid) rates. (Right) Computed Orbach transition rates, τ^–1^, between energy states for **2** at 100 K. Larger values of τ^–1^ indicate more probable transitions.

To further analyze the relaxation mechanism and identify the phonon modes that most significantly influence the dynamics, we calculate τ with selective inclusion of phonon modes below a target high-energy cutoff frequency ω_max_ or above a low-energy cutoff frequency ω_min_. We fix the simulation temperature at 20 K for Raman processes and 100 K for Orbach processes, the results are reported in Figure 6 for Raman and Orbach mechanisms, respectively. Considering first the Orbach mechanism (Figure 6, left), a sharp change in the computed relaxation time is only observed when ω_max_ approaches the energy gap between the first and second Kramers doublets, around 400 cm^–1^. This highlights the role of an initial transition from the ground state to the first excited Kramers doublet in the relaxation pathway of these compounds. By examining the behavior of τ as a function of ω_min_, we observe that an almost complete quenching of the relaxation is achieved only when phonon modes with energies above the position of the third Kramers doublet (around 800 cm^–1^) are excluded. This piece of evidence illustrates two interesting facts. First, the slow growth of relaxation time by removing phonons with smaller energy than the first excited KD points to an active role of higher-energy KDs in the relaxation pathway in the high-temperature (100 K) used to perform the analysis. Second, this analysis suggests that multiple relaxation pathways with similar efficiency are possible. Indeed, only when phonons with an energy of around 800 cm^–1^ are removed, we do observe an abrupt change in relaxation time, meaning that the relaxation pathway involving a direct absorption to the second excited KD is not dramatically less efficient than the one proceeding single steps. The role of the third Kramers doublet in driving the relaxation is further corroborated by the evaluation of the effective relaxation barrier *U*_eff_ from the fitting of the Orbach relaxation times measured across a temperature range of 65–70 K. The fitted value is ∼638 cm^–1^ for both compounds, and it increases to 714 cm^–1^ at 100 K.

**Figure 6:**
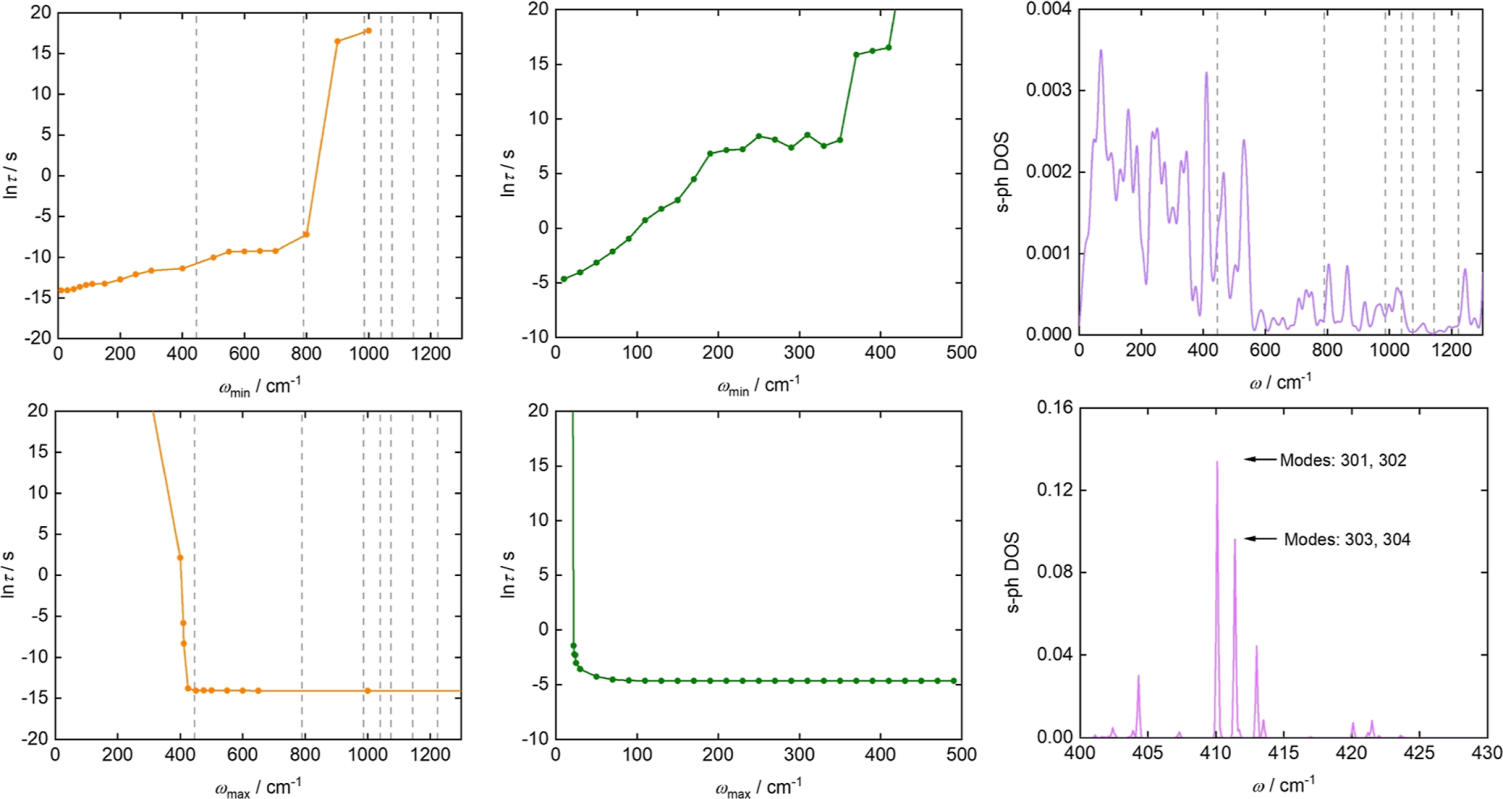
(Left) Orbach contribution for **2** to τ as a function of low-energy cutoff ω_min_ (top) and as a function of high-energy cutoff ω_max_ (bottom). Temperature is fixed at 100 K. Kramers doublet energy levels are reported as dashed lines. (Middle) Raman contribution for **2** to τ as a function of low-energy cutoff ω_min_ (top) and as a function of high-energy cutoff ω_max_ (bottom). Temperature is fixed at 20 K. (Right) Spin–phonon coupling density of states *D*(ω) of **2** (top). The analyzed modes are indicated in the *s*-ph DOS plot (bottom). The groups of atoms primarily involved in the vibrations of modes 301, 302, 303, and 304 are circled in the molecular geometry (Supporting Information, Figure S47).

In contrast, in the Raman regime (Figure 6, middle), the rapid convergence of τ as a function of the high-energy cutoff ω_max_ demonstrates that low-energy phonons are primarily responsible for Raman relaxation. This conclusion is further supported by the trend observed when τ is plotted against the low-energy cutoff ω_min_: the relaxation time increases sharply with increasing ω_min_, indicating that excluding low-energy phonons significantly quenches the relaxation process. A visual inspection of the low-energy phonon modes 10 and 11, located near 22 cm^–1^, reveals that they are strongly delocalized across the unit cell, with large contributions from rigid motions of the helicene ligands, as well as the noncoordinated BPh_4_^–^ and CH_2_Cl_2_ molecules (see Movies S1, S2, S3, S4, and S5 provided in the Supporting Information). The low-energy phonons responsible for the Raman relaxation process contributed cannot therefore easily be associated with localized molecular features but instead receive contributions from weak packing interactions.

Finally, to identify the nature of the main vibrational modes that contribute to the Orbach relaxation, we analyze the spin–phonon coupling density of states *D*(ω), defined as

6 $$D\left(\omega \right)=\sum_{\alpha }\sum_{lm}{\left(\frac{\partial {\mathrm{B̂}}_{m}^{l}}{\partial Q_{\alpha }}\right)}^{2}\delta \left(\omega -\omega_{a}\right)$$

Results are reported in the right panel of Figure 6. A large intensity peak is found in the vicinity of 400 cm^–1^, where the previous analysis locates the high-energy cutoff for which the relaxation via the third Kramers doublet occurs (Figure 6, left). The most strongly coupled phonons have been identified by further resolving *D*(ω) around 400 cm^–1^. Eigenmodes numbered 301, 302, 303, and 304 are the most strongly coupled in this energy region. All these modes have a similar character and involve vibrations of the equatorial benzene rings and the two nearest nitrogen atoms, as illustrated in Figure S49. The animation of mode 301 is attached to this document (“mod 301.mpg”).

To conclude the analysis of relaxation pathways, we also consider the low-*T* regime dominated by QTM. The modelization of such a relaxation mechanism from first-principles is still in its infancy, but several computational approaches have been proposed in recent years to describe this regime.^61,62^ Here, we adopted the method introduced by Yin et al. based on correlating QTM to the size of the transverse components of the effective *g*-tensor of the ground-state KD.^63^ Using this approach, we obtain a QTM relaxation time of τ_QTM_ = 0.05 s, in good agreement with the experimental results.

## Conclusion

In summary, we report the design, synthesis, SMM behaviors, and spin dynamics of the first lanthanide–[7]­helicene compounds. By exploiting the high tunability in axial positions of chiral lanthanide hexaazamacrocycle, we have developed a new strategy to prepare multichiral lanthanide–helicene species. In this Dy­(III) derivate, the dominant interaction between Dy­(III) and axial [7]­helicene ligand generates strong magnetic anisotropy, which ensures the magnetic bistability and induces high-temperature magnetic relaxation. Both *U*_eff_ value and hysteresis temperature far surpass all reported lanthanide–helicene SMMs. Looking forward to further extending the magnetic relaxation time for this novel class of molecules, ab initio spin dynamics calculations revealed that (i) the vibrations of two phenyl substituents in the chiral diamine units at 400 cm^–1^ are strongly coupled to spin and resonant with the second Kramers doublet, thus very effectively driving the first step of relaxation that occur via the third Kramers doublet; and (ii) it is the low-energy phonons that mainly account for Raman relaxation.

The results indicate that there remains scope to increase the blocking temperature and coercivity by enhancing the rigidity of both the molecular structures and their crystal packing. A promising strategy to achieve this could be ring-closing metathesis (RCM) between adjacent phenyl substituents, which would restrict molecular motion and thereby suppress detrimental vibrational modes. In addition to this, owing to their intrinsic chirality and strong magnetic anisotropy, both compounds are promising candidates for observing MChD and the spin-electric effect (SEE).^64,65^ The open hysteresis loops at zero field provide a crucial basis for subsequent detection of hysteresis using unpolarized light.^66^ Furthermore, the superposition of helical and point chirality in these molecules is anticipated to lead to an enhanced chirality-transfer efficiency, making them potentially valuable for applications in enantioselective synthesis.^67^

## Supplementary Material













## Notes

Experimental details and crystal and magnetic property data (PDF)
(MP4)
(MP4)
(MP4)
(MP4)
(MP4)
