# Supplementary material for: Chiral Dysprosium-[7]Helicene Macrocycles Showing Record Single-Molecule Magnet Properties in the Lanthanide–Helicene Family
Source: J Am Chem Soc. 2025 Nov 10;147(46):42815–24. doi: 10.1021/jacs.5c15088 (PMC12636023; doi:10.1021/jacs.5c15088)
Supplement: Supplementary file 1 [file ja5c15088_si_001.pdf]

Supporting Information for

Chiral Dysprosium-[7]Helicene Macrocycles Showing Record Single-Molecule Magnet Properties in Lanthanide-Helicene Family

Zhenhua Zhu,[a,#,*] Tingting Wang,[a,b,#] Lorenzo A. Mariano,[c] Sagar Paul,[d] Wolfgang Wernsdorfer,[d,e] Alessandro Lunghi[c,*] and Jinkui Tang[a,b,*]

[a]State Key Laboratory of Rare Earth Resource Utilization, Changchun Institute of Applied Chemistry, Chinese Academy of Sciences, Changchun 130022, P. R. China

[b]School of Applied Chemistry and Engineering, University of Science and Technology of China, Hefei 230026, P. R. China

[c]School of Physics, AMBER and CRANN Institute, Trinity College, Dublin 2, Ireland

[d]Physikalisches Institut, Karlsruhe Institute of Technology (KIT), Karlsruhe D-76131, Germany

[e]Institute for Quantum Materials and Technology (IQMT), Karlsruhe Institute of Technology (KIT), Eggenstein-Leopoldshafen D-76344, Germany

*Email: zhuzh@ciac.ac.cn; lunghia@tcd.ie; tang@ciac.ac.cn

[#]Z. Z. and T. W. contributed equally to this work.

Contents

1. Synthesis and characterization S3
2. X-ray crystallography data S22
3. Magnetic measurements S287
4. Theoretical calcualtions S39
5. References S43

1. Synthesis and characterization

General procedure

Unless otherwise noted, all oxygen or moisture sensitive reactions were conducted in dried glassware under an atmosphere of nitrogen or argon. THF was purified and dried according to standard method. Other solvents and reagents purchased from commercial sources were used without further purification. All coordination reaction manipulations described were performed under aerobic conditions. 2-Bromo[5]helicene (**S5**) has been synthesized in five steps from Benzaldehyde according to the published method.[1-2] A 500 W high-pressure mercury lamp was used for photoreactions. Thin-layer Chromatography (TLC) was performed on aluminum sheets precoated with 0.25 mm thick silica gel GF254 (Shanghai Shengya Chemicals (China)) and visualized by exposure to ultraviolet light. Chromatographic purification of products was accomplished using forced-flow chromatography on 200-300 mesh silica gel.

Measurements

^{1}H NMR spectra were recorded on Bruker Avance 400 MHz spectrometer. Chemical shifts are reported in parts per million relative to the residual proton signal of the solvent $CDCl_3$ (7.26 ppm) and DMSO (2.50 ppm). Elemental analyses (C, H, N) were performed on a Perkin-Elmer 2400 analyzer. FT-IR spectra were recorded with a Nicolet 6700 Flex FTIR spectrometer equipped with a smart iTR attenuated total reflectance (ATR) sampling accessory in the range from 4000 to 535 cm^{-1}. UV-Vis spectra (220 – 470 nm) were performed on UV-1750 UV-Vis spectrophotometer and the solution circular dichroism (CD) spectra (230 – 450 nm) were measured on a Jasco J-820 spectropolarimeter and Jasco J-1500 spectropolarimeter at room temperature using Hellma high precision cell of 10 mm light path length. Mass spectrum (MS) was recorded on a Thermo Fisher LTQ XL Linear Ion Trap Mass Spectrometer using electrospray ionization (ESI). HPLC were performed on a CHIRALPAK ID (250 × 4.6 mm, 5 μm) in hexane:*i*-PrOH 95:5, flow rate 1.0 ml/min, simultaneous UV detection at 254 nm. Thermogravimetric analyses were performed on a Netzsch STA449F3 TG-DSC instrument in the range of 30-800 ºC with a heating rate of 10 K min^{-1} under the N_2 condition. Direct current (DC) magnetic susceptibility and magnetization data were recorded on a Quantum Design MPMS-XL7 SQUID magnetometer equipped with a 7 T magnet. Variable-temperature magnetic susceptibility ware collected with an external magnetic field of 1000 Oe in the temperature range of 2-300 K. Magnetic hysteresis loops were collected using a Quantum Design MPMS3 magnetometer in warming mode. Alternating current (AC) magnetic susceptibility measurements were performed using a Quantum Design MPMS3 magnetometer using an oscillating field of 2 Oe. The experimental magnetic susceptibility data were corrected for the diamagnetism estimated from Pascal's tables and sample holder calibration.[3] Note that all complexes can rapidly lose DCM from the lattice at room temperature. Therefore, all measurements were performed after the sample had been exposed to air for several days at room temperature.

Synthesis

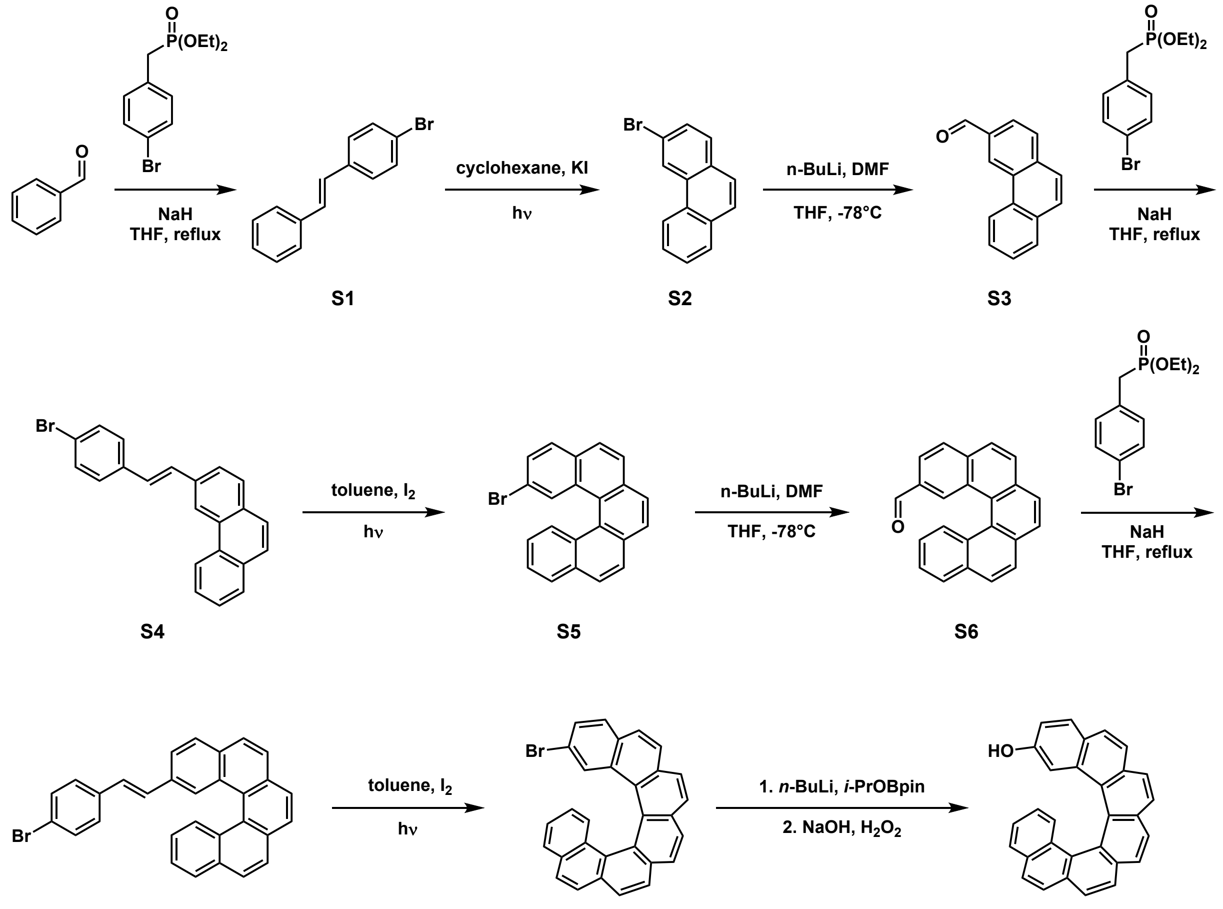

Scheme S1. Synthetic routes of **HL**.

Synthetic procedure for (*E*)-1-bromo-4-styrylbenzene (**S1**)

A round-bottom flask was charged with sodium hydride (60% in mineral oil, 6.22 g, 155.5 mmol, 1.1 equiv) under N_2 and dry THF (160 mL) was added. Into the suspension, diethyl 4-bromobenzylphosphonate (47.80 g, 155.5 mmol, 1.1 equiv) was added dropwise and stirred for 40 min. Benzaldehyde (14.4 ml, 141.4 mmol, 1 equiv) was dissolved in dry THF (20 mL) and added dropwise. The mixture was heated up to 70 °C and stirred for 24 h. The resulting mixture was quenched with water (400 mL). The mixture was filtered and the residue was washed with *n*-pentane, giving a white solid (34.0 g, 131.2 mmol, 93% yield) as the desired product **S1**. **^{1}H NMR** (400 MHz, $CDCl_3$) δ (ppm):

7.49 (dd, *J* = 10.8, 8.0 Hz, 4H), 7.42 - 7.33 (m, 4H), 7.29 (d, *J* = 7.3 Hz, 1H), 7.14 - 7.00 (m, 2H).

Synthetic procedure for 3-bromophenanthrene (**S2**)

(*E*)-1-bromo-4-styrylbenzene (**S1**) (2.40 g, 9.3 mmol, 1 equiv) was dissolved in 4.5 L of cyclohexane and potassium iodide (1.54 g, 9.3 mmol) was added. The mixture obtained was irradiated under stirring for 9 h with a Hg lamp (500 W). The solvent was evaporated under reduced pressure, and the residue was dissolve in dichloromethane. The solution was washed with aqueous sodium hydrogen sulfite ($NaHSO_3$) (100 ml) and brine, dried over anhydrous Na_2SO_4, and filtered, and the solvent was evaporated under reduced pressure. The crude residue was purified by column chromatography on

silica gel (eluent: petroleum ether) to afford compound **S2** (1.21 g, 4.7 mmol, 50% yield) as a white solid. **^{1}H NMR** (400 MHz, $CDCl_3$) δ (ppm): 8.82 (s, 1H), 8.60 (d, *J* = 8.1 Hz, 1H), 7.89 (d, *J* = 7.8 Hz, 1H), 7.76 (d, *J* = 8.6 Hz, 2H), 7.73 - 7.59 (m, 4H).

Synthetic procedure for phenanthrene-3-carbaldehyde (**S3**)
3-bromophenanthrene (**S2**) (13.00 g, 50.6 mmol, 1 equiv) was dissolved in dry tetrahydrofuran (100 mL) under N_2 atmosphere and cooled down to -78 °C. *n*-BuLi (2.4 M, 27.4 mL, 65.7 mmol, 1.3 equiv) was added dropwise and the mixture was stirred at -78 °C for an hour. Afterward, *N,N*-dimethylformamide (10.1 mL, 131.4 mmol, 2.6 equiv) was added dropwise and the reaction was stirred for additional 1 h. The resulting mixture was warmed up to room temperature, excessive *n*-BuLi was quenched by MeOH (15 mL) and H_2O (30 ml) sequentially, reaction mixture was extracted with dichloromethane (3 × 20 mL), and combined organic layers were dried over anhydrous Na_2SO_4 and concentrated under reduced pressure. The crude residue was purified by flash column chromatography on silica gel (eluent: petroleum ether/EtOAc = 40/1) to afford compound **S3** (7.63 g, 36.9 mmol, 73% yield) as a white solid. **^{1}H NMR** (400 MHz, $CDCl_3$) δ (ppm): 10.24 (s, 1H), 9.12 (s, 1H), 8.74 (d, *J* = 8.2 Hz, 1H), 8.05 (dd, *J* = 8.2, 1.4 Hz, 1H), 7.96 (d, *J* = 8.2 Hz, 1H), 7.94 - 7.85 (m, 2H), 7.78 - 7.63 (m, 3H).

Synthetic procedure for (*E*)-3-(4-bromostyryl)phenanthrene (**S4**)
A round-bottom flask was charged with sodium hydride (60% in mineral oil, 1.56 g, 38.9 mmol, 1.1 equiv) under N_2 and dry THF (80 mL) was added. Into the suspension, diethyl 4-bromobenzylphosphonate (11.96 g, 38.9 mmol, 1.1 equiv) was added dropwise and stirred for 20 min. Phenanthrene-3-carbaldehyde (**S3**) (7.30 g, 35.4 mmol, 1 equiv) was dissolved in dry THF (20 mL) and added dropwise. The mixture was heated up to 70 °C and stirred for 24 h. The resulting mixture was quenched with water (400 mL). The mixture was filtered and the residue was washed with *n*-pentane, giving a pale yellow solid (12.47 g, 34.7 mmol, 98% yield) as the desired product **S4**. **^{1}H NMR** (400 MHz, DMSO) δ (ppm): 9.01 (s, 1H), 8.91 (d, *J* = 8.2 Hz, 1H), 7.99 (t, *J* = 7.0 Hz, 3H), 7.84 (s, 2H), 7.77 - 7.70 (m, 1H), 7.66 (dd, *J* = 16.4, 4.8 Hz, 5H), 7.58 (d, *J* = 6.4 Hz, 2H).

Synthetic procedure for 2-Bromo[5]helicene (**S5**)
(*E*)-3-(4-bromostyryl)phenanthrene (**S4**) (2.40 g, 6.7 mmol, 1 equiv) was dissolved in 4.5 L of toluene and iodine (140 mg, 0.6 mmol) was added. The mixture obtained was irradiated under stirring for 9 h with a Hg lamp (500 W). After the reaction, excess iodine was quenched by adding a saturated aqueous solution of sodium thiosulfate ($Na_2S_2O_3$) (100 ml). The organic phase was separated and concentrated under reduced pressure. The crude residue was purified by flash column chromatography on silica gel (eluent: petroleum ether/DCM = 50/1) to afford compound **S5** (1.68 g, 4.7 mmol, 70% yield) as a yellow solid. **^{1}H NMR** (400 MHz, $CDCl_3$) δ (ppm): 8.70 (s, 1H), 8.52 (d, *J* = 8.5 Hz, 1H), 8.01 - 7.92 (m, 2H), 7.91 - 7.83 (m, 5H), 7.81 (d, *J* = 8.6 Hz, 1H), 7.63 - 7.53 (m, 2H), 7.40 - 7.32 (m, 1H).

Synthetic procedure for 2-carbaldehyde[5]helicene (**S6**)
2-Bromo[5]helicene (**S5**) (4.50 g,12.6 mmol, 1 equiv) was dissolved in dry tetrahydrofuran (120 mL) under N_2 atmosphere and cooled down to -78 °C. *n*-BuLi (2.4 M, 6.8 mL, 16.4 mmol, 1.3 equiv) was added dropwise and the mixture was stirred at -78 °C for an hour. Afterward, *N,N*-dimethylformamide

(2.5 mL, 32.7 mmol, 2.6 equiv) was added dropwise and the reaction was stirred for additional 1 h. The resulting mixture was warmed up to room temperature, excessive *n*-BuLi was quenched by MeOH (15 mL) and H_2O (30 ml) sequentially, reaction mixture was extracted with dichloromethane (3 × 20 mL), and combined organic layers were dried over anhydrous Na_2SO_4 and concentrated under reduced pressure. The crude residue was purified by flash column chromatography on silica gel (eluent: petroleum ether/DCM = 10/1 to petroleum ether/EtOAc = 30/1) to afford compound **S6** (2.60 g, 8.5 mmol, 66% yield) as a pale yellow solid. **^{1}H NMR** (400 MHz, DMSO) δ (ppm): 9.77 (s, 1H), 8.93 (s, 1H), 8.32 (d, *J* = 8.5 Hz, 1H), 8.24 (d, *J* = 8.8 Hz, 2H), 8.20 - 8.05 (m, 6H), 7.98 (d, *J* = 8.3 Hz, 1H), 7.61 (t, *J* = 7.4 Hz, 1H), 7.31 (t, *J* = 7.7 Hz, 1H).

Synthetic procedure for (*E*)-9-(4-bromostyryl)dibenzo[*c,g*]phenanthrene (**S7**)
A Round bottom flask was charged with sodium hydride (60%, 0.37 g, 9.3 mmol, 1.1 equiv) under N_2 atmosphere and dry THF (95 mL) was added. Into the suspension, diethyl (4-bromobenzyl)phosphonate (2.87 g, 9.3 mmol) was added dropwise and stirred for 30 min. 2-carbaldehyde[5]helicene (**S6**) (2.60 g, 8.5 mmol) was dissolved in dry THF (15 mL) and added dropwise. The mixture was heated up to 70 °C and stirred for 24 h. The resulting mixture was quenched with water (200 mL). The mixture was filtered and the residue was washed with *n*-pentane, giving a yellow solid (3.52 g, 7.6 mmol, 90% yield) as the desired product **S7**. **^{1}H NMR** (400 MHz, DMSO) δ (ppm): 8.51 (s, 1H), 8.43 (d, *J* = 8.6 Hz, 1H), 8.07 (dt, *J* = 22.9, 8.5 Hz, 8H), 7.88 (d, *J* = 8.4 Hz, 1H), 7.61 (t, *J* = 7.5 Hz, 1H), 7.52 (d, *J* = 8.4 Hz, 2H), 7.42 (d, *J* = 8.4 Hz, 2H), 7.32 (t, *J* = 7.2 Hz, 1H), 7.17 - 6.99 (m, 2H).

Synthetic procedure for 2-Bromo[7]helicene (**S8**)
(*E*)-9-(4-bromostyryl)dibenzo[*c,g*]phenanthrene (**S7**) (1.75 g, 3.8 mmol, 1 equiv) was dissolved in 4.5 L of toluene and iodine (100 mg, 0.8 mmol) was added. The mixture obtained was irradiated under stirring for 9 h with a Hg lamp (500 W). After the reaction, excess iodine was quenched by adding a saturated aqueous solution of sodium thiosulfate ($Na_2S_2O_3$) (100 ml). The organic phase was separated and concentrated under reduced pressure. The crude residue was purified by flash column chromatography on silica gel (eluent: petroleum ether/ Et_2O = 5/1 to petroleum ether/DCM = 5/1) to afford compound **S8** (0.81 g, 1.8 mmol, 46% yield) as a yellow solid. **^{1}H NMR** (400 MHz, $CDCl_3$) δ (ppm): 8.09 - 7.98 (m, 5H), 7.92 (d, *J* = 8.2 Hz, 1H), 7.83 (d, *J* = 8.5 Hz, 1H), 7.76 (d, *J* = 8.5 Hz, 1H), 7.62 (d, *J* = 8.5 Hz, 1H), 7.45 (d, *J* = 8.5 Hz, 1H), 7.38 (d, *J* = 8.0 Hz, 1H), 7.31 (d, *J* = 1.7 Hz, 1H), 7.17 (d, *J* = 8.5 Hz, 1H), 7.12 (d, *J* = 8.5 Hz, 1H), 7.00 (dd, *J* = 8.5, 1.9 Hz, 1H), 6.95 - 6.89 (m, 1H), 6.40 (ddd, *J* = 8.3, 6.9, 1.3 Hz, 1H).

Synthetic procedure for 2-Hydroxy[7]helicene (**HL**)
2-Bromo[7]helicene (**S8**) (3.10 g, 6.8 mmol, 1 equiv) was dissolved in dry tetrahydrofuran (120 mL) under N_2 atmosphere and cooled down to -78 °C. *n*-BuLi (2.4 M, 3.7 mL, 8.8 mmol, 1.3 equiv) was added dropwise and the mixture was stirred at -78 °C for half an hour. Afterward, isopropoxyboronate pinacol ester (1.8 mL, 8.8 mmol, 1.3 equiv) was added dropwise and the reaction was slowly allowed to warm up to room temperature and stirred for an additional 60 min. The reaction was quenched with diluted HCl (0.5 mL, 10% V/V aqueous solution) and evaporated to dryness. The residuum was dissolved in THF (30 mL) and was added NaOH (1.28 g, 31.9 mmol, 6.0 equiv), and an aqueous

solution of H_2O_2 (30%, 3.3 mL, 31.9 mmol, 6.0 equiv) was added dropwise. The reaction was stirred for 30 min at room temperature and extracted with EtOAc (15 × 3 mL), washed with water (30 mL), brine (30 mL), dried over anhydrous Na_2SO_4 and filtered. The solvents were removed at the reduced pressure and the crude product was purified by flash chromatography on silica gel (eluent: petroleum ether/EtOAc = 10/1) to afford compound **HL** (1.03 g, 2.6mmol, 39%) as a yellow solid. **^{1}H NMR** (400 MHz, $CDCl_3$) δ (ppm): 8.05 - 7.90 (m, 6H), 7.77 (d, *J* = 8.5 Hz, 1H), 7.64 (d, *J* = 8.4 Hz, 2H), 7.45 (d, *J* = 8.4 Hz, 1H), 7.39 (d, *J* = 7.9 Hz, 1H), 7.23 (d, *J* = 8.6 Hz, 1H), 7.14 (d, *J* = 8.5 Hz, 1H), 6.97 - 6.90 (m, 1H), 6.57 (dd, *J* = 8.6, 2.5 Hz, 1H), 6.48 (d, *J* = 2.4 Hz, 1H), 6.42 (ddd, *J* = 8.3, 7.0, 1.2 Hz, 1H), 3.91 (s, 1H). ESI-MS (*m*/*z*): [M]- calcd. for C30H17O, 393.47; found, 393.0.

Synthetic procedure for **1**

2,6-diformylpyridine (6.8 mg, 0.05 mmol) and (1*R*, 2*R*)-1,2- diphenylethylenediamine (10.6 mg, 0.05 mmol) was added to a methanol solution (5 mL) of $DyCl_3$□$6H_2O$ (9.4 mg, 0.025 mmol). The reaction mixture was stirred for 5 h under refluxing. After cooled down to room temperature, the methanol solvent was removed and the white solid was collected as a chiral macrocyclic precursor.[4] The precursor was dissolved in a mixture solvent of 10 mL DCM and 10 mL deionized water. Subsequently, **HL** (0.1 mmol, 39.5 mg), triethylamine (0.1 mmol, 14 μl) and $NaBPh_4$ (0.025 mmol, 8.6 mg) were added to the mixture and then the mixture was heated to reflux for 20 min. After cooled down to room temperature, DCM phase was separated and filtered. Orange-red crystals of **1** suitable for X-ray diffraction were isolated by layering DCM phase with n-pentane at 25 °C after five days, affording a reproducible yield (10.0 mg, 21% based on Dy). Elemental analysis (%) calcd for $C_{126}H_{88}BDyN_6O_2$ (M_W = 1891.44): C, 80.01; H, 4.64; N, 4.44. Found: C, 79.78; H, 4.75; N, 4..43. FTIR *ν*/cm^{-1} (ATR): 3044 (w), 1650 (w), 1592 (m), 1487 (w), 1424 (m), 1337 (m), 1285 (m), 1266 (m), 1230 (m), 1162 (m), 1054 (m), 1006 (m), 921 (m), 839 (vs), 729 (m), 696 (s), 611 (w) , 592 (w), 568 (w).

Synthetic procedure for **2**

The synthetic procedure of **2** is similar to **1**, except that (1S, 2S)- 1,2- diphenylethylenediamine was used rather than (1R, 2R)- 1,2- diphenylethylenediamine. Yield = 14.0 mg, (29% based on Dy). Elemental analysis (%) calcd for $C_{126}H_{88}BDyN_6O_2$ (M_W = 1891.44): C, 80.01; H, 4.64; N, 4.44. Found: C, 79.84; H, 4.84; N, 4.42. FTIR *ν*/cm^{-1} (ATR): 3044 (w), 1650 (w), 1592 (m), 1488 (w), 1424 (m), 1338 (m), 1285 (m), 1267 (m), 1231 (m), 1163 (m), 1054 (m), 1006 (m), 921 (m), 839 (vs), 729 (w), 696 (s), 611 (w) , 592 (w), 568 (w).

Synthetic procedure for ***P*-HL**

To a stirred solution of **1** (10.0 mg, 5.3 μmol) in DCM (10 mL) was added diluted HCl (10 mL, 10% V/V aqueous solution). The mixture was stirred at room temperature for 30 min. The organic phase was separated and washed with deionized water (3 × 8 mL), then concentrated under reduced pressure. The crude product was purified by column chromatography on silica gel (eluent: DCM) to afford chiral compound ***P*-HL** (2.0 mg, 5.0 μmol, 95% yield, 97.1% ee) as a pale yellow solid.

Synthetic procedure for ***M*-HL**

The synthetic procedure of ***M*-HL** is similar to ***P*-HL**, except that complex **2** was used rather than complex **1**. Yield = 2.0 mg, (5.1 μmol, 97% yield, 96.5% ee)

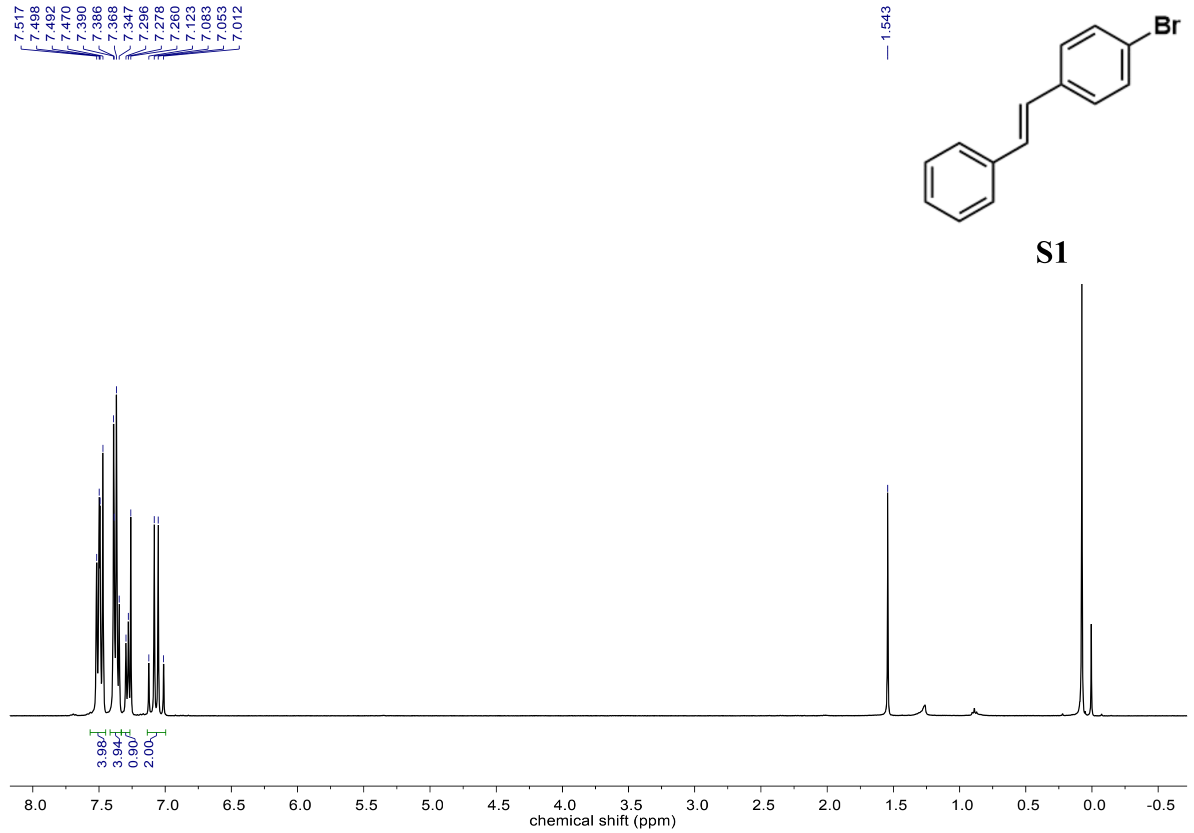

Figure S1. ^{1}H-NMR (400 MHz) spectrum of compound **S1** in $CDCl_3$.

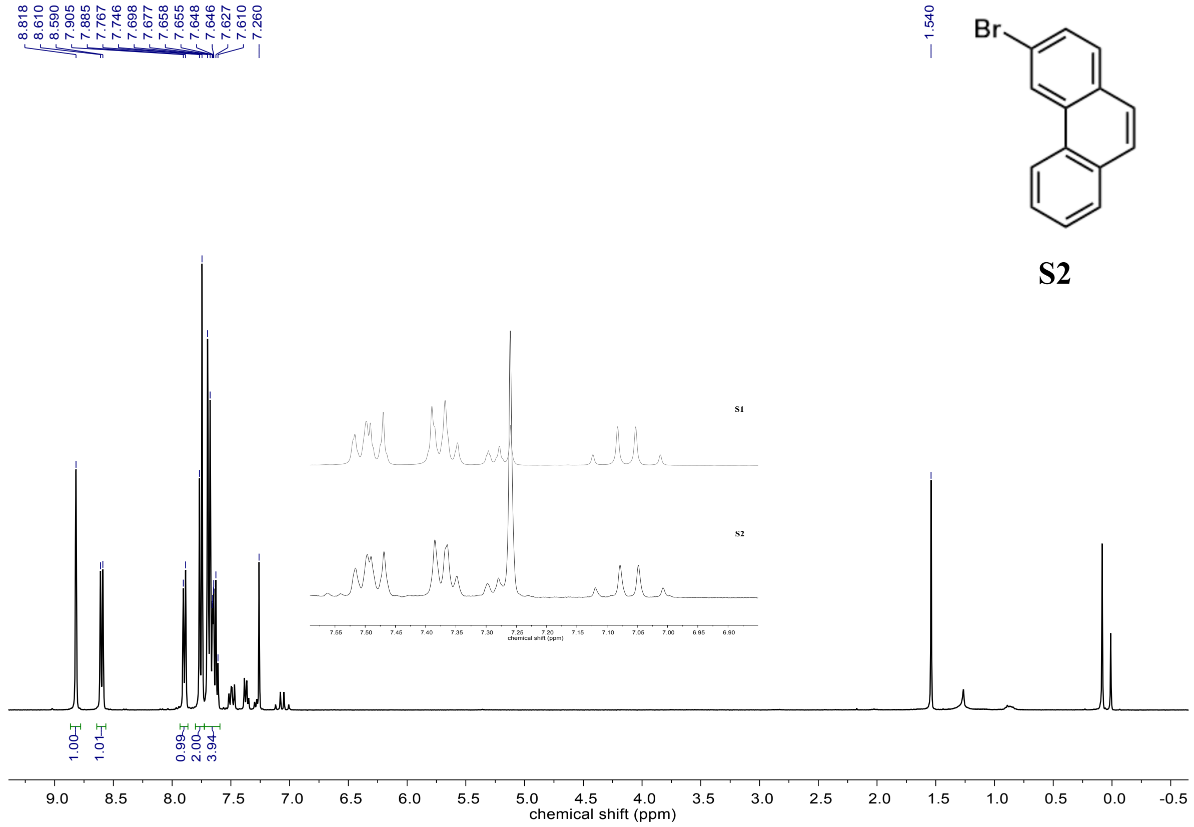

Figure S2. ^{1}H-NMR (400 MHz) spectrum of compound **S2** in $CDCl_3$. The signals in 6.95 - 7.55 ppm are assigned to the compound **S1**.

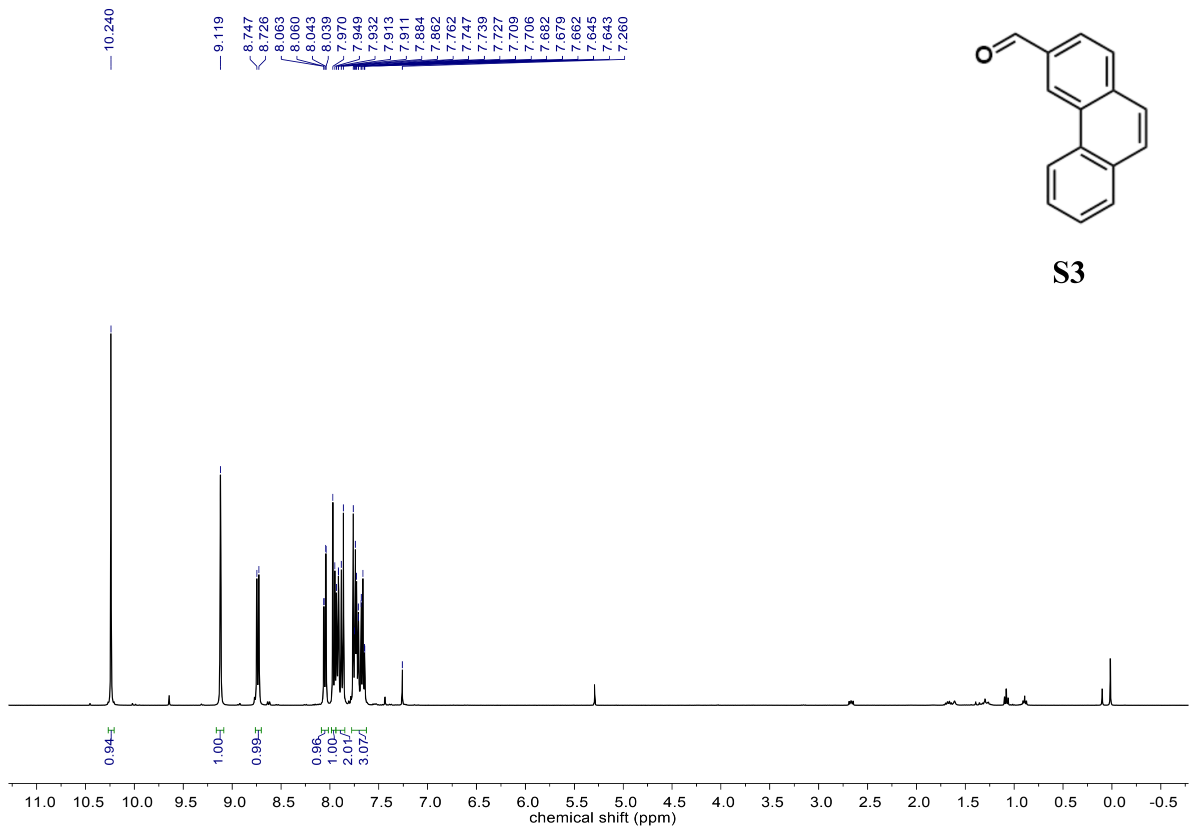

Figure S3. ^{1}H-NMR (400 MHz) spectrum of compound **S3** in $CDCl_3$.

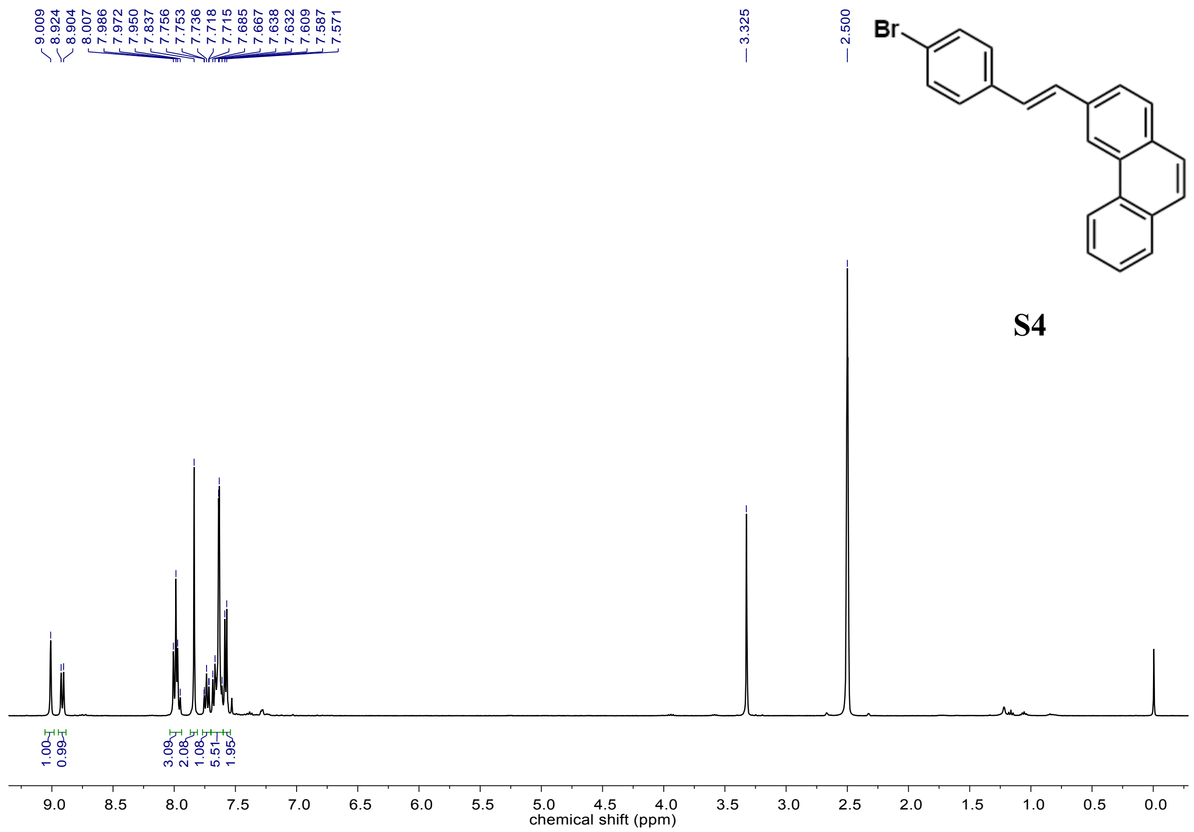

Figure S4. ^{1}H-NMR (400 MHz) spectrum of compound **S4** in DMSO.

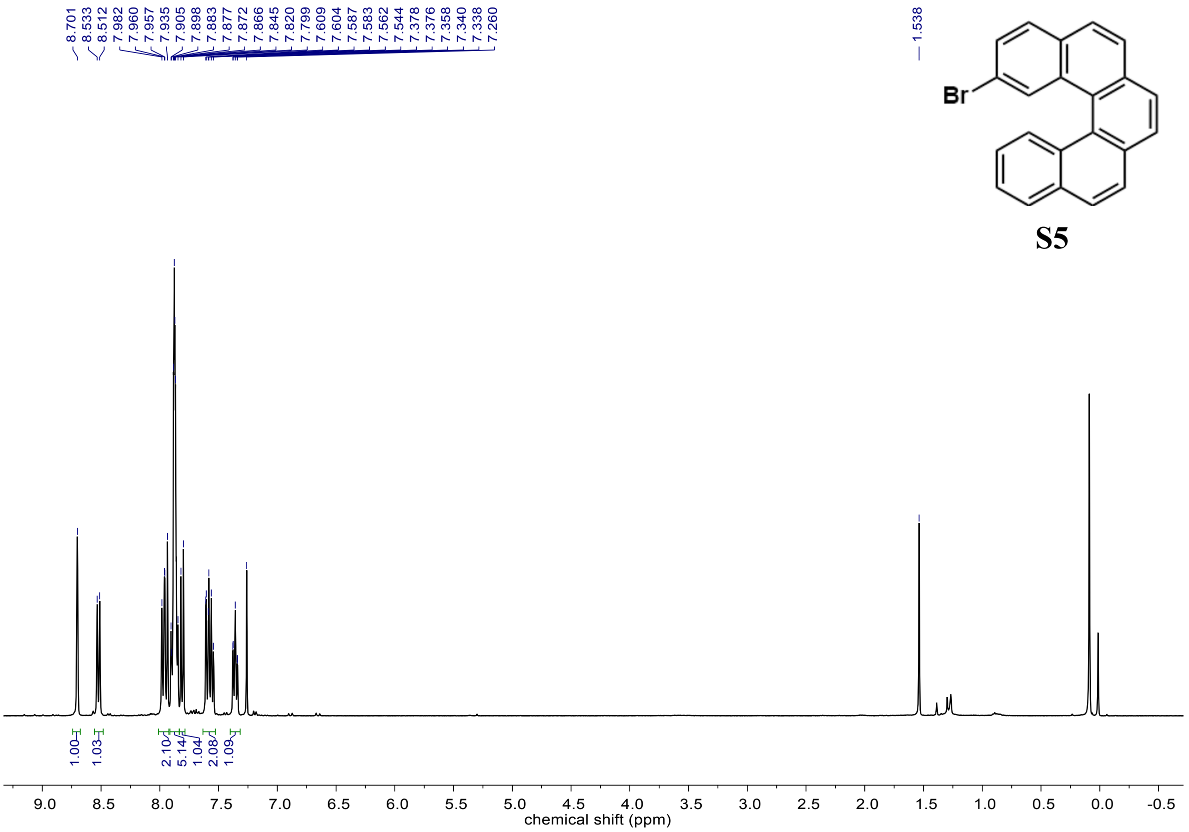

Figure S5. ^{1}H-NMR (400 MHz) spectrum of compound **S5** in $CDCl_3$.

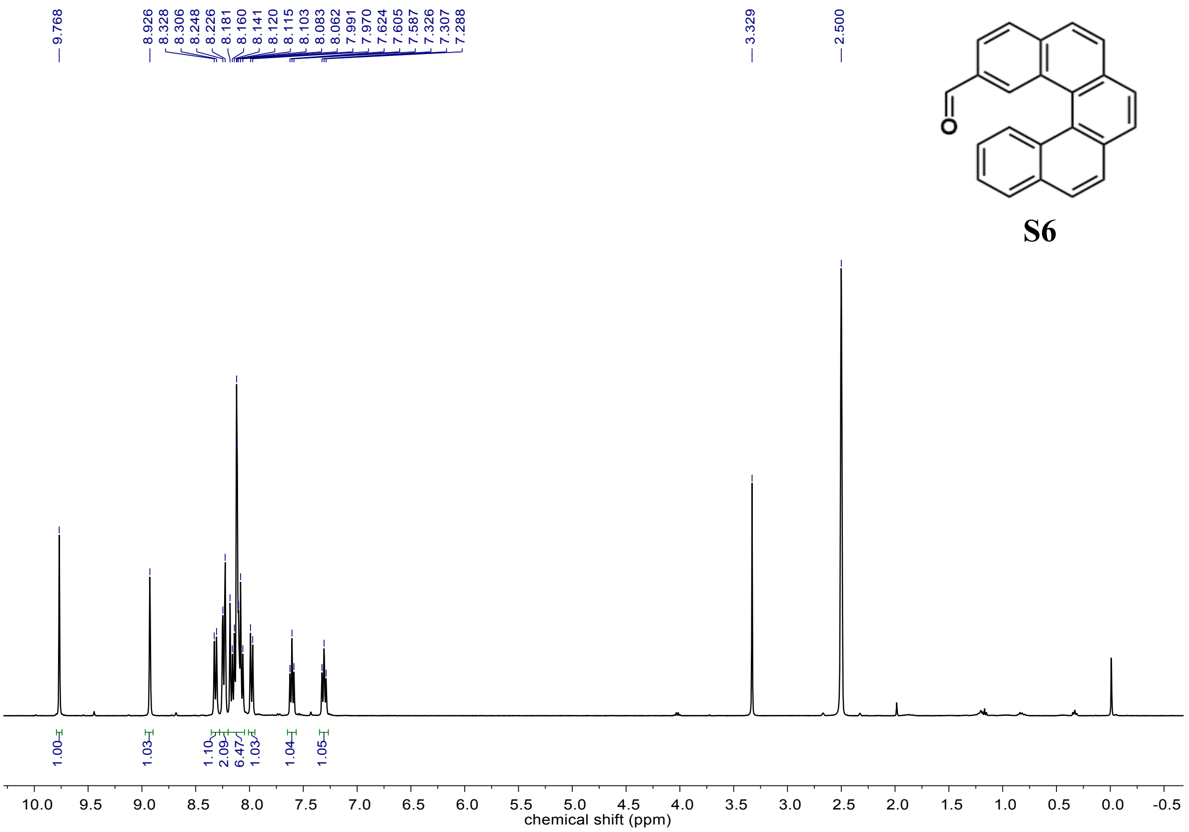

Figure S6. ^{1}H-NMR (400 MHz) spectrum of compound **S6** in DMSO.

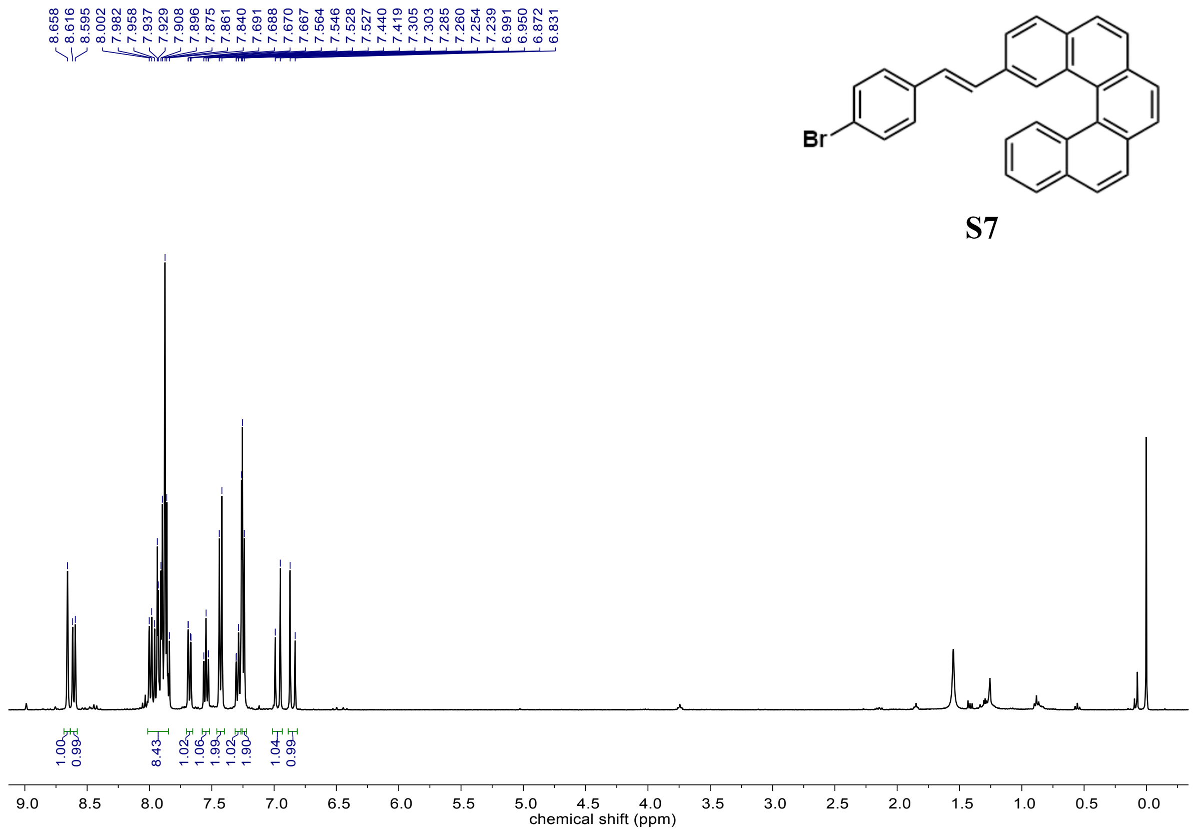

Figure S7. ^{1}H-NMR (400 MHz) spectrum of compound **S7** in DMSO.

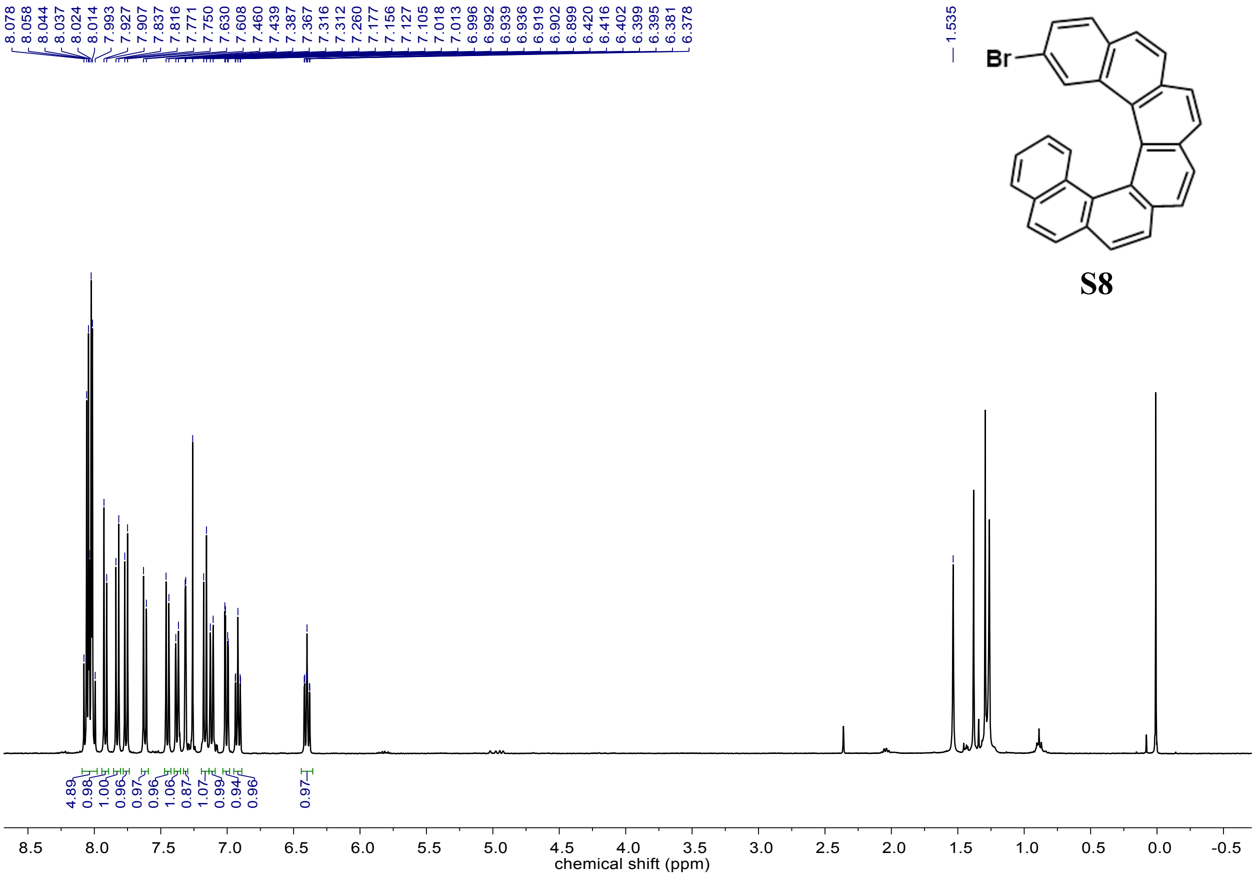

Figure S8. ^{1}H-NMR (400 MHz) spectrum of compound **S8** in $CDCl_3$.

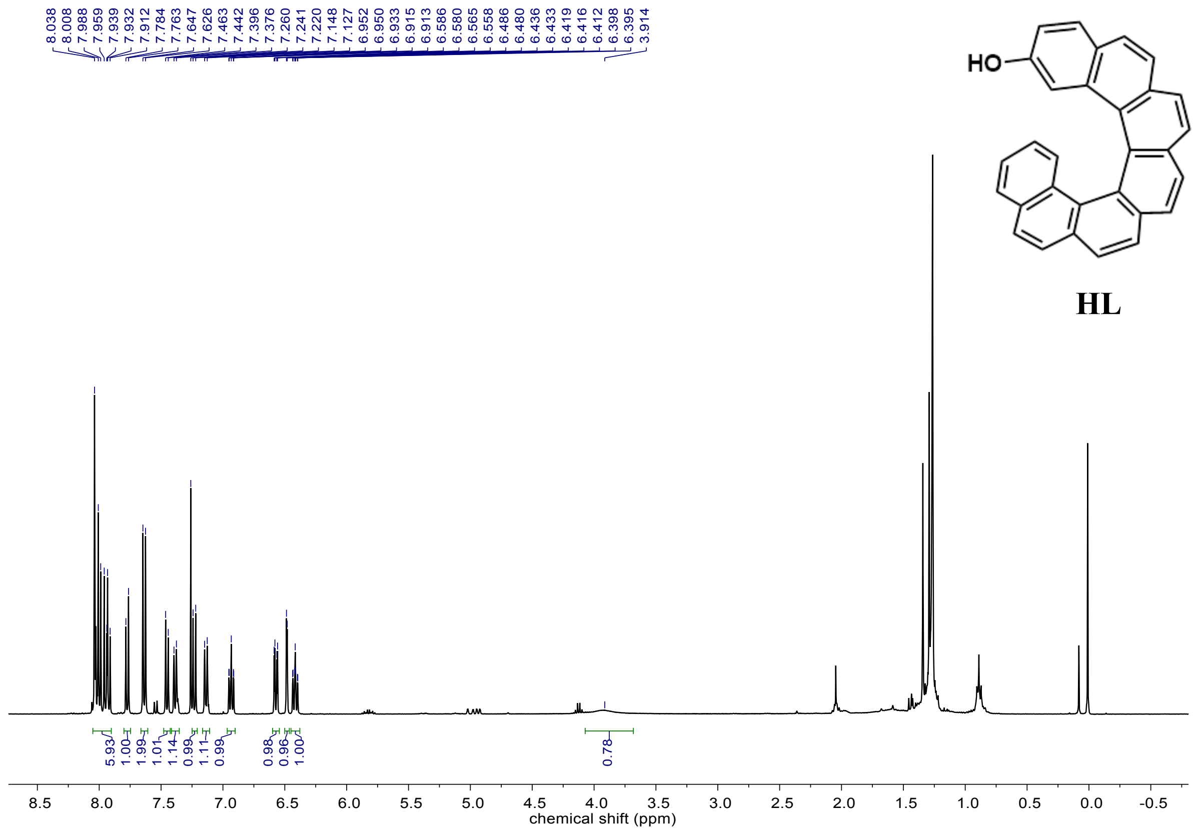

Figure S9. ^{1}H-NMR (400 MHz) spectrum of compound **HL** in $CDCl_3$.

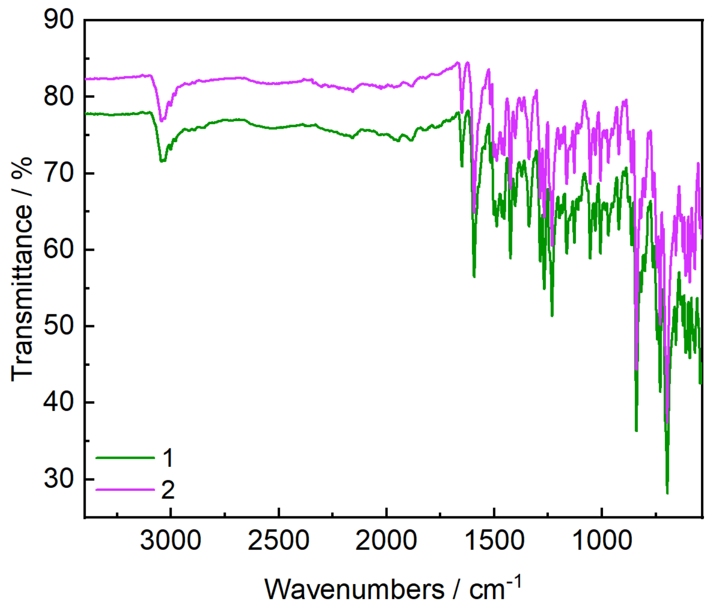

Figure S10. FT-IR (ATR) spectra of solid samples for complex **1** and **2**.

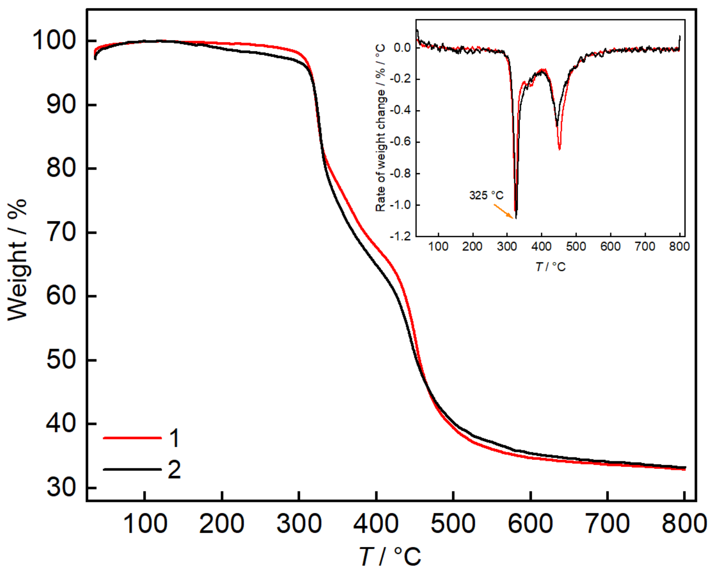

Figure S11. Thermogravimetric analysis of **1** (red line) and **2** (black line).

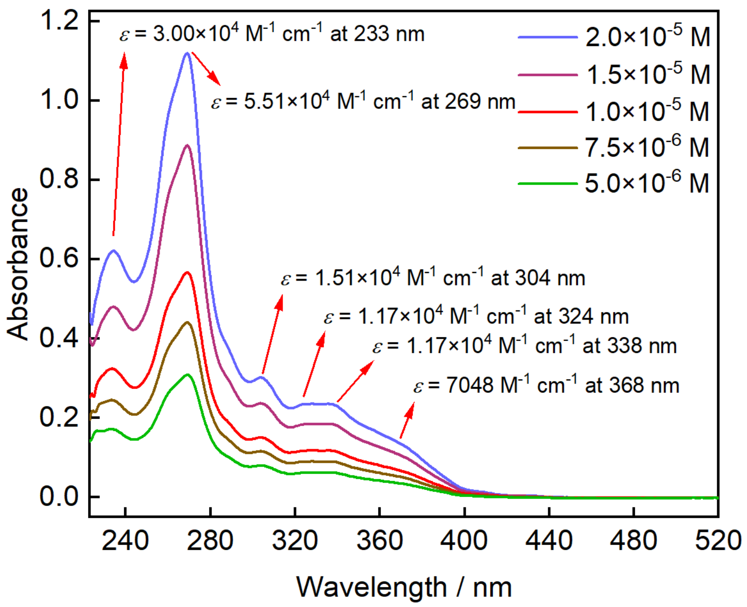

Figure S12. UV-vis spectrum of **HL** in CH_2Cl_2 at varying concentrations.

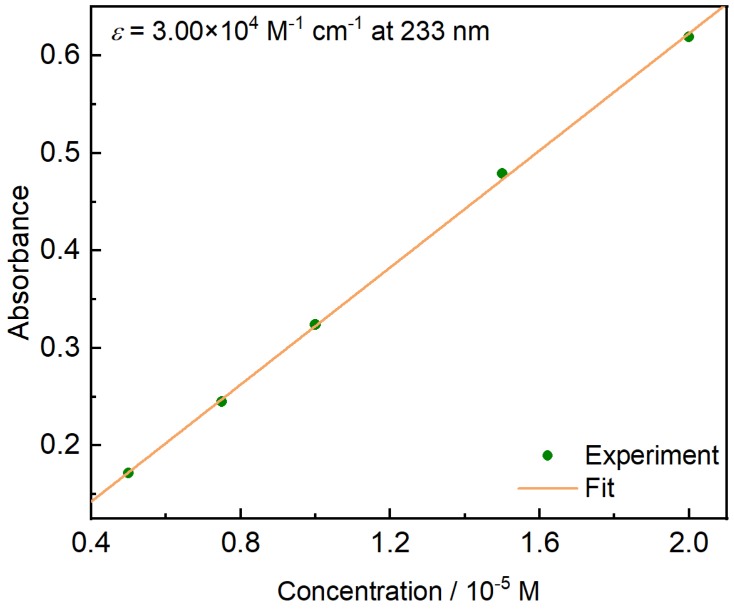

Figure S13. Plot of absorbance versus concentration for **HL** at 233 nm in the UV-vis spectrum. The green points are from the UV-vis spectrum (Figure S11). The solid orange line is the best fit to the data to extract the extinction coefficient.

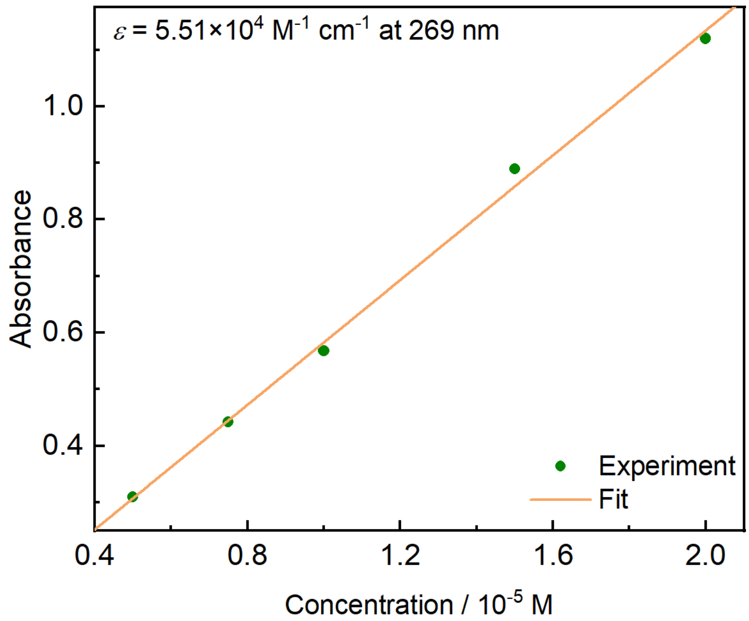

Figure S14. Plot of absorbance versus concentration for **HL** at 269 nm in the UV-vis spectrum. The green points are from the UV-vis spectrum (Figure S11). The solid orange line is the best fit to the data to extract the extinction coefficient.

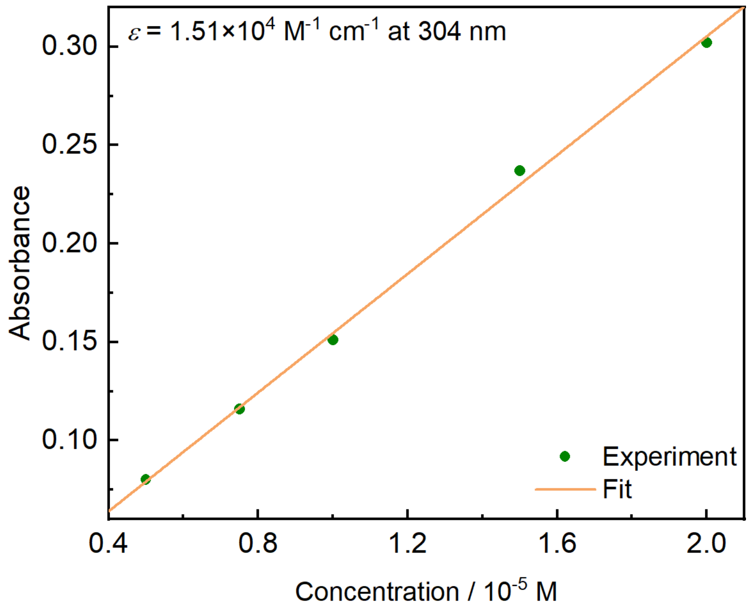

Figure S15. Plot of absorbance versus concentration for **HL** at 304 nm in the UV-vis spectrum. The green points are from the UV-vis spectrum (Figure S11). The solid orange line is the best fit to the data to extract the extinction coefficient.

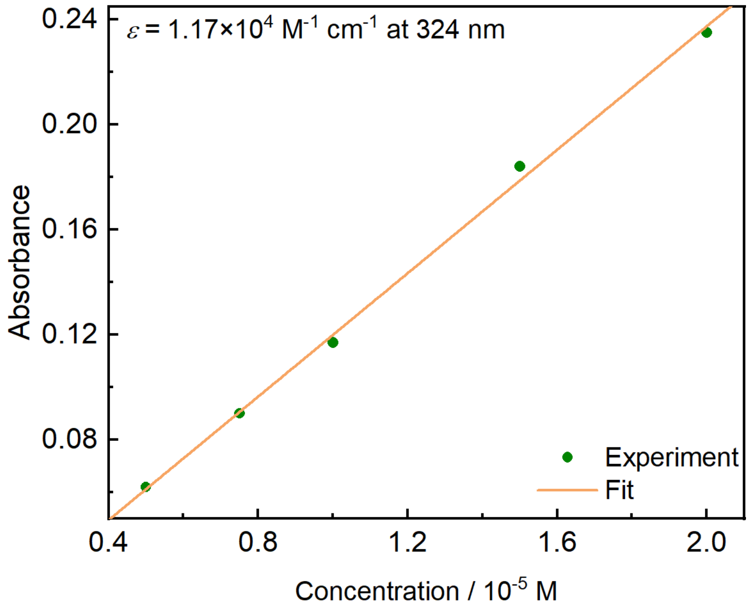

Figure S16. Plot of absorbance versus concentration for **HL** at 324 nm in the UV-vis spectrum. The green points are from the UV-vis spectrum (Figure S11). The solid orange line is the best fit to the data to extract the extinction coefficient.

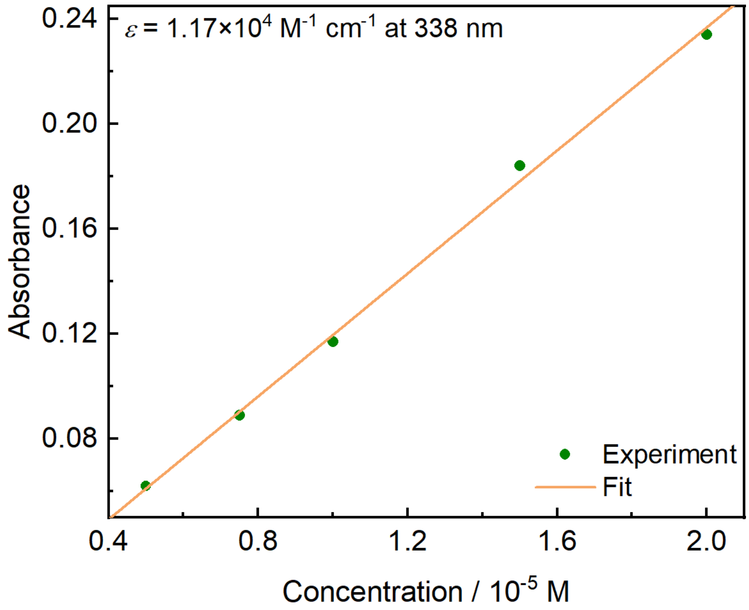

Figure S17. Plot of absorbance versus concentration for **HL** at 338 nm in the UV-vis spectrum. The green points are from the UV-vis spectrum (Figure S11). The solid orange line is the best fit to the data to extract the extinction coefficient.

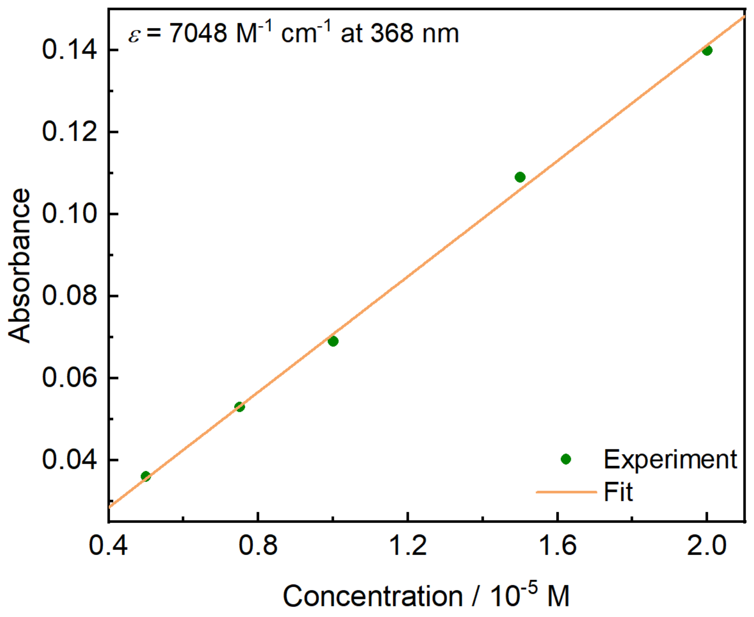

Figure S18. Plot of absorbance versus concentration for **HL** at 368 nm in the UV-vis spectrum. The green points are from the UV-vis spectrum (Figure S11). The solid orange line is the best fit to the data to extract the extinction coefficient.

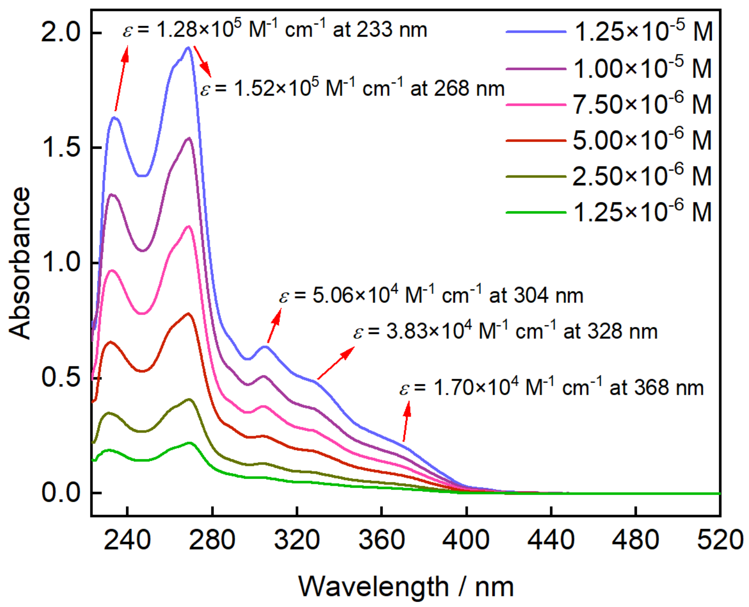

Figure S19. UV-vis spectrum of **1** in CH_2Cl_2 at varying concentrations.

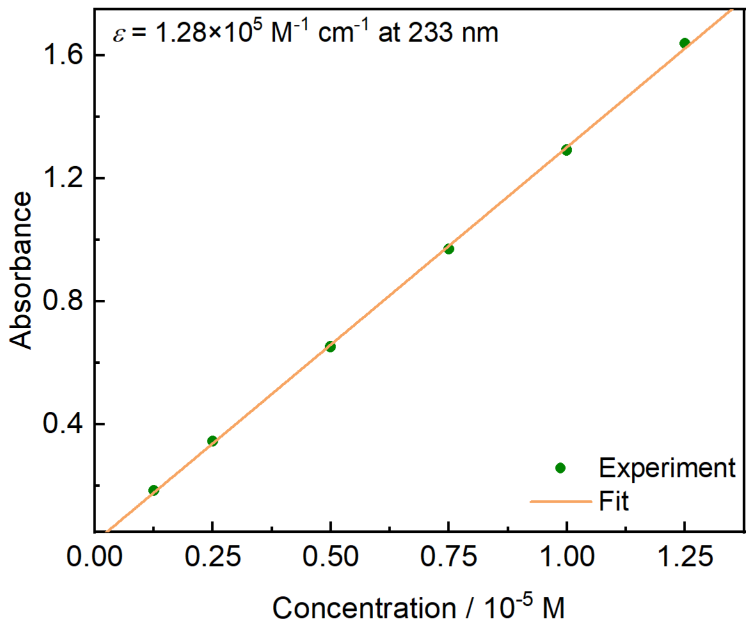

Figure S20. Plot of absorbance versus concentration for **1** at 233 nm in the UV-vis spectrum. The green points are from the UV-vis spectrum (Figure S18). The solid orange line is the best fit to the data to extract the extinction coefficient.

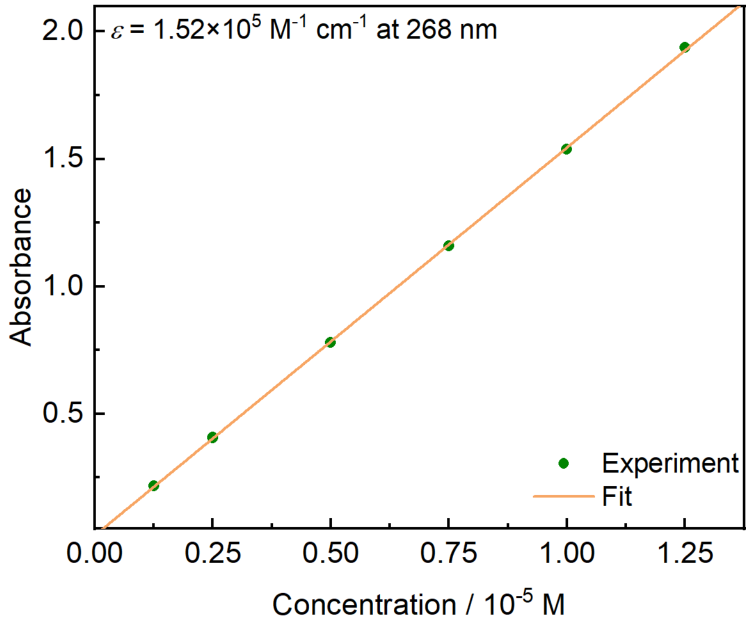

Figure S21. Plot of absorbance versus concentration for **1** at 268 nm in the UV-vis spectrum. The green points are from the UV-vis spectrum (Figure S18). The solid orange line is the best fit to the data to extract the extinction coefficient.

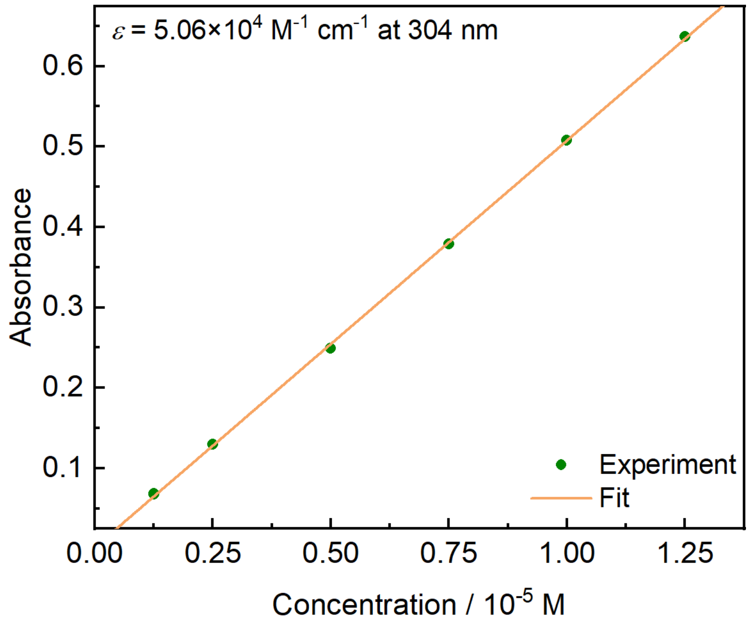

Figure S22. Plot of absorbance versus concentration for **1** at 304 nm in the UV-vis spectrum. The green points are from the UV-vis spectrum (Figure S18). The solid orange line is the best fit to the data to extract the extinction coefficient.

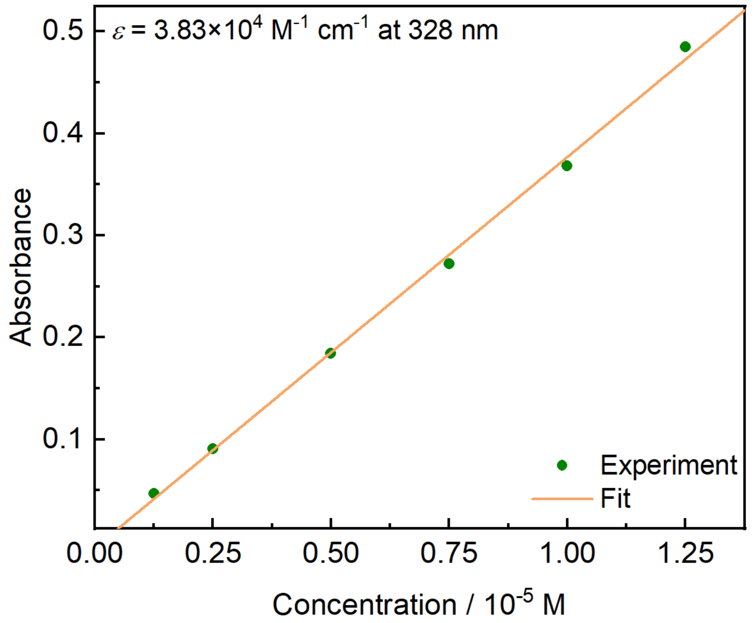

Figure S23. Plot of absorbance versus concentration for **1** at 328 nm in the UV-vis spectrum. The green points are from the UV-vis spectrum (Figure S18). The solid orange line is the best fit to the data to extract the extinction coefficient.

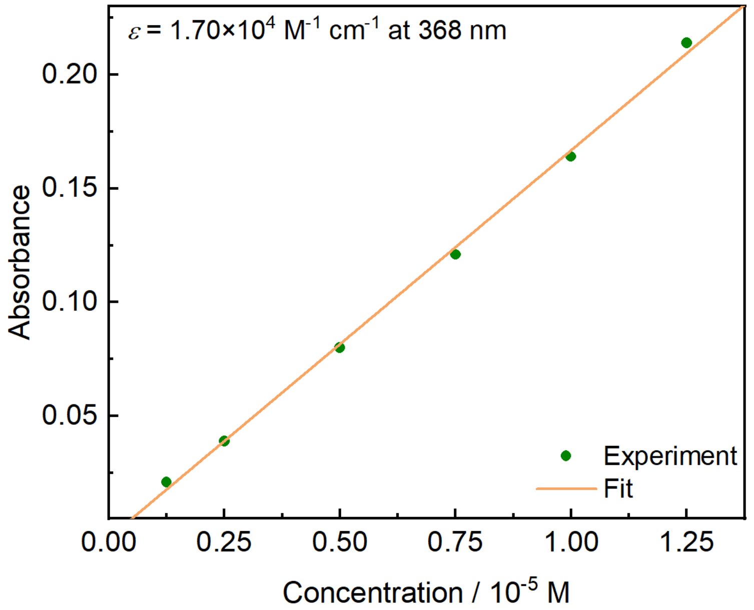

Figure S24. Plot of absorbance versus concentration for **1** at 368 nm in the UV-vis spectrum. The green points are from the UV-vis spectrum (Figure S18). The solid orange line is the best fit to the data to extract the extinction coefficient.

2. X-ray crystallography data

Single-crystal X-ray data for **1** and **2** were recorded on a Bruker SMART APEX diffractometer equipped with graphite-monochromatized Mo Kα radiation (λ = 0.71073 Å) at 180 K. The structures were solved in Olex2 with SHELXT using intrinsic phasing and were refined with SHELXL using least squares minimization.[5-7] All non-hydrogen atoms were refined anisotropically. All hydrogen atom positions were calculated geometrically and refined using the riding model. Crystallographic data, refinement details are given in Tables S1-S3.

Table S1. Crystal data and structure refinement for **1** and **2**.

**Compound reference**	**1**	**2**
Chemical formula	$C_{129}H_{94}BCl_6DyN_6O_2$	$C_{129}H_{94}BCl_6DyN_6O_2$
Formula Mass	2146.11	2146.11
Crystal system	monoclinic	monoclinic
a (Å)	17.5879(9)	17.5169(10)
b (Å)	14.4343(6)	14.4758(7)
c (Å)	20.7108(9)	20.6842(11)
α (°)	90	90
β (°)	94.164(2)	93.896(2)
γ (°)	90	90
Unit cell volume (Å^3)	5244.0(4)	5232.8(5)
Temperature (K)	180.0	180.0
Space group	$P2_1$	$P2_1$
Z	2	2
ρ_{calc} (g/cm^3)	1.359	1.362
F (000)	2198.0	2198.0
Radiation	MoKα (λ = 0.71073)	MoKα (λ = 0.71073)
Reflections collected	46248	63713
Independent reflections	18445	18463
R_{int}	0.0890	0.1154
GOF on F^2	1.032	1.038
R_1 (I ≥2 σ (I))	0.0584	0.0555
wR_2 (all data)	0.1456	0.1186
Flack parameter	-0.005(7)	-0.005(6)
CCDC number	2457487	2457488

Table S2. Selected bond distances (Å) for **1** and **2**.

1	2
Dy1-O1 2.106(8)	Dy1-O1 2.099(8)
Dy1-O2 2.131(8)	Dy1-O2 2.129(7)
Dy1-N1 2.656(9)	Dy1-N1 2.613(9)
Dy1-N2 2.661(9)	Dy1-N2 2.658(8)
Dy1-N3 2.608(10)	Dy1-N3 2.667(8)
Dy1-N4 2.679(10)	Dy1-N4 2.619(9)
Dy1-N5 2.683(8)	Dy1-N5 2.683(8)
Dy1-N6 2.608(9)	Dy1-N6 2.682(8)

Table S3. Selected bond angles (°) for **1** and **2**.

1	2
O1-Dy1-O2 165.8(3)	O1-Dy1-O2 165.3(3)
O1-Dy1-N1 92.7(3)	O1-Dy1-N1 86.6(2)
O1-Dy1-N2 106.3(3)	O1-Dy1-N2 105.9(3)
O1-Dy1-N3 86.5(3)	O1-Dy1-N3 92.7(3)
O1-Dy1-N4 77.6(3)	O1-Dy1-N4 100.5(3)
O1-Dy1-N5 83.3(3)	O1-Dy1-N5 82.8(3)
O1-Dy1-N6 100.3(3)	O1-Dy1-N6 77.4(3)
O2-Dy1-N1 98.1(3)	O2-Dy1-N1 96.6(3)
O2-Dy1-N2 87.1(3)	O2-Dy1-N2 88.1(3)
O2-Dy1-N3 96.4(3)	O2-Dy1-N3 98.4(3)
O2-Dy1-N4 91.5(3)	O2-Dy1-N4 76.8(2)
O2-Dy1-N5 83.4(3)	O2-Dy1-N5 83.3(3)
O2-Dy1-N6 77.3(3)	O2-Dy1-N6 91.5(3)
N1-Dy1-N2 59.9(3)	N1-Dy1-N2 61.0(3)
N1-Dy1-N4 170.3(3)	N1-Dy1-N4 172.8(3)
N1-Dy1-N5 119.3(3)	N1-Dy1-N5 121.6(3)
N2-Dy1-N4 121.7(3)	N2-Dy1-N4 115.2(3)
N2-Dy1-N5 170.3(3)	N2-Dy1-N5 171.2(2)
N3-Dy1-N1 118.1(3)	N3-Dy1-N1 118.0(3)
N3-Dy1-N2 61.1(3)	N3-Dy1-N2 59.8(2)
N3-Dy1-N4 61.2(3)	N3-Dy1-N4 60.9(3)
N3-Dy1-N5 122.0(3)	N3-Dy1-N5 119.7(2)
N4-Dy1-N5 60.9(3)	N4-Dy1-N5 61.1(3)
N6-Dy1-N1 61.0(3)	N6-Dy1-N1 61.4(3)
N6-Dy1-N2 115.3(3)	N6-Dy1-N2 121.9(2)
N6-Dy1-N3 173.1(3)	N6-Dy1-N3 170.1(2)
N6-Dy1-N4 121.1(3)	N6-Dy1-N4 121.1(3)
N6-Dy1-N5 60.4(3)	N6-Dy1-N5 60.2(2)

Table S4. The CShM values calculated by SHAPE 2.1 for **1** and **2**.[8-9]

Central atom	Coordination Geometry	**1**	**2**
Dy	Octagon (D_{8h})	28.641	28.560
	Heptagonal pyramid (C_{7v})	18.888	18.867
	Hexagonal bipyramid (D_{6h})	2.009	2.064
	Cube (O_h)	7.520	7.557
	Square antiprism (D_{4d})	13.253	13.290
	Triangular dodecahedron (D_{2d})	11.193	11.217
	Johnson gyrobifastigium J26 (D_{2d})	7.889	7.968
	Johnson elongated triangular bipyramid J14 (D_{3h})	23.577	23.599
	Biaugmented trigonal prism J50 (C_{2v})	12.754	12.810
	Biaugmented trigonal prism (C_{2v})	12.779	12.793
	Snub diphenoid J84 (D_{2d})	13.747	13.811
	Triakis tetrahedron (T_d)	8.380	8.418
	Elongated trigonal bipyramid (D_{3h})	22.010	22.004

Table S5. Dihedral angle and interplanar angle in [7]helicene, HL and *P*-L in **1**.

Geometric parameters		[7]helicene	HL	*P*-L in **1**
Dihedral angles /°	C1-C2-C3-C4	17.412	10.583	12.919
	C2-C3-C4-C5	24.742	24.548	25.458
	C3-C4-C5-C6	24.405	28.451	27.496
	C4-C5-C6-C7	23.904	23.646	24.783
	C5-C6-C7-C8	18.792	15.264	13.612
Interplanar angle /°		32.388	46.311	45.448

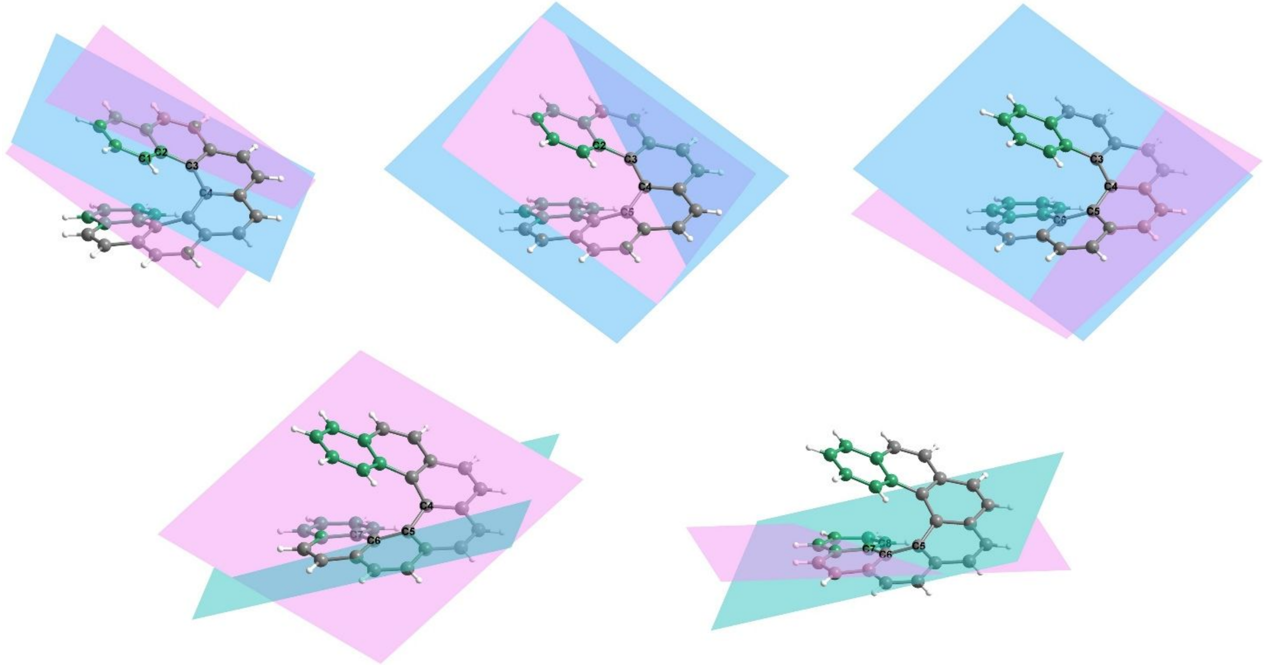

Figure S25. The torsional angles in [7]-helicene with *P*-helicity.

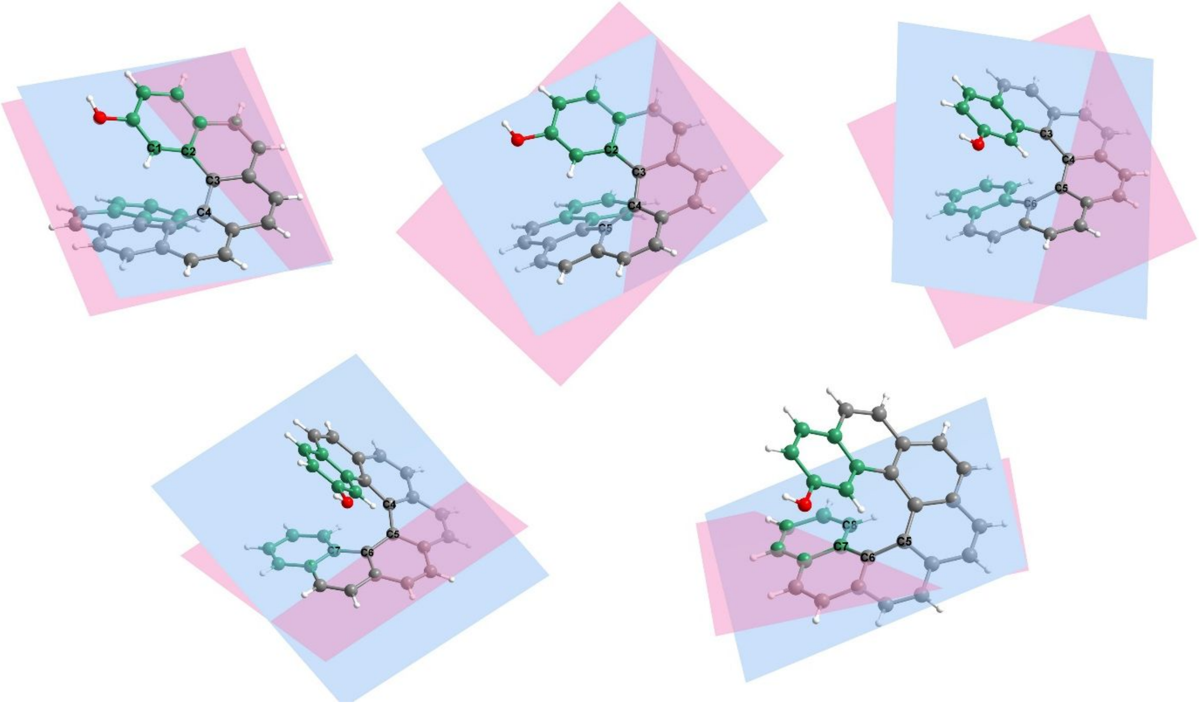

Figure S26. The torsional angles in HL.

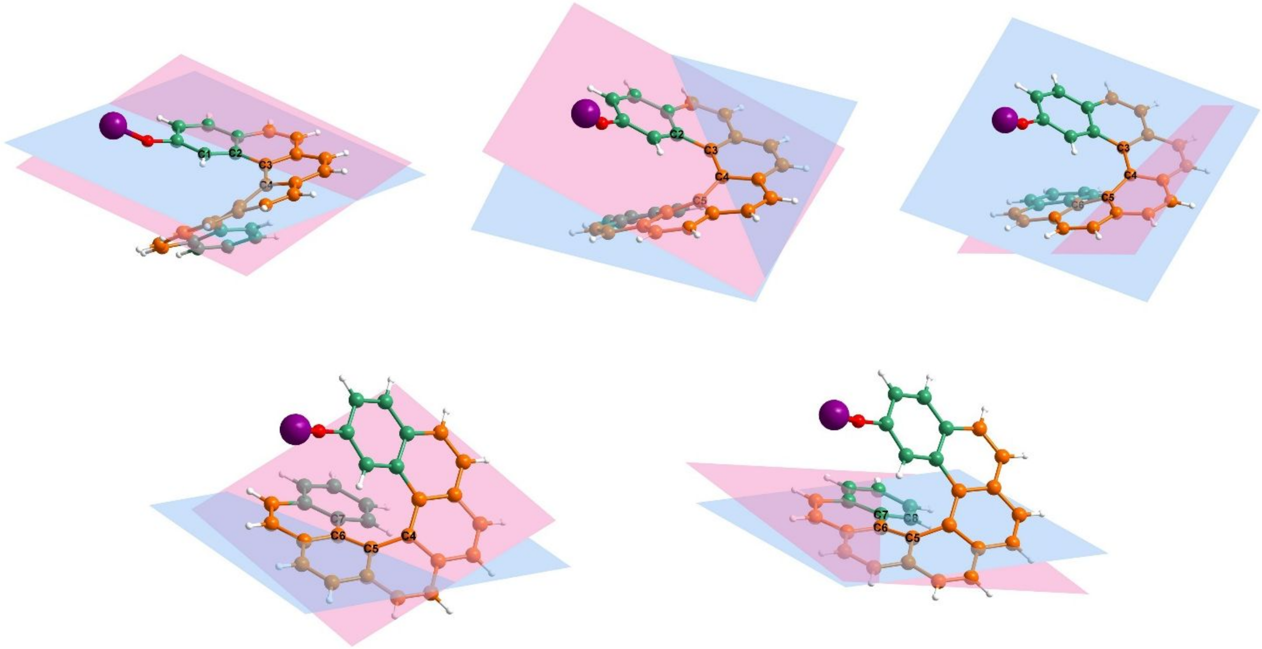

Figure S27. The torsional angles in *P*-L in compound **1**.

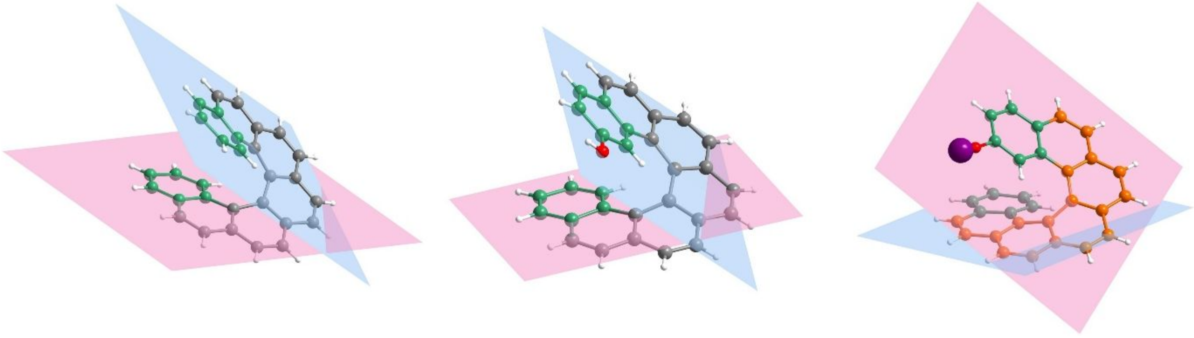

Figure S28. The interplanar angles in P-[7]helicene, HL and *P*-L in compound **1** (from left to right).

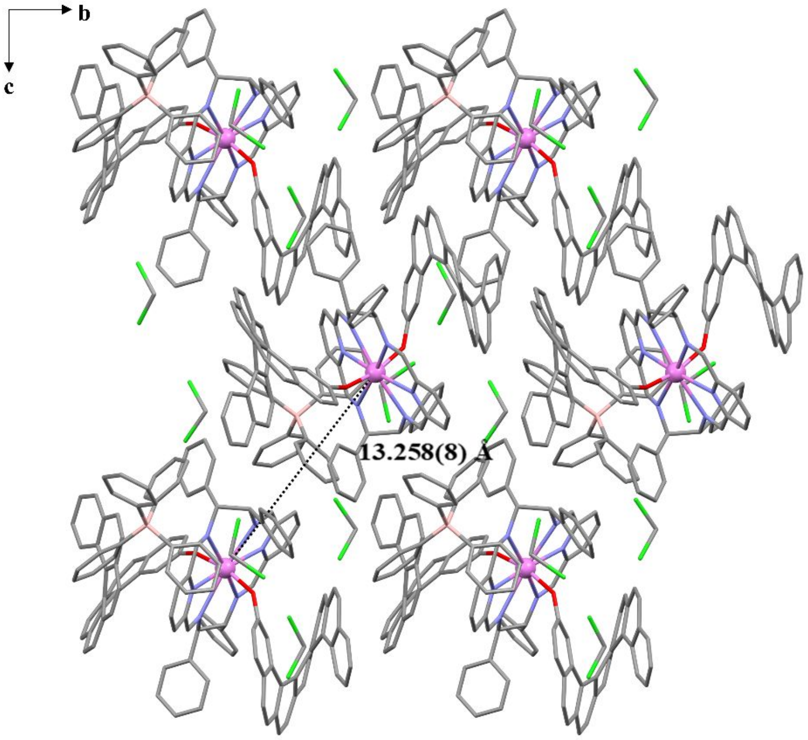

Figure S29. The packing diagram for **1** shown along the crystallographic *a* axis gives the shortest intermolecular Dy···Dy distance of 13.258(8) Å.

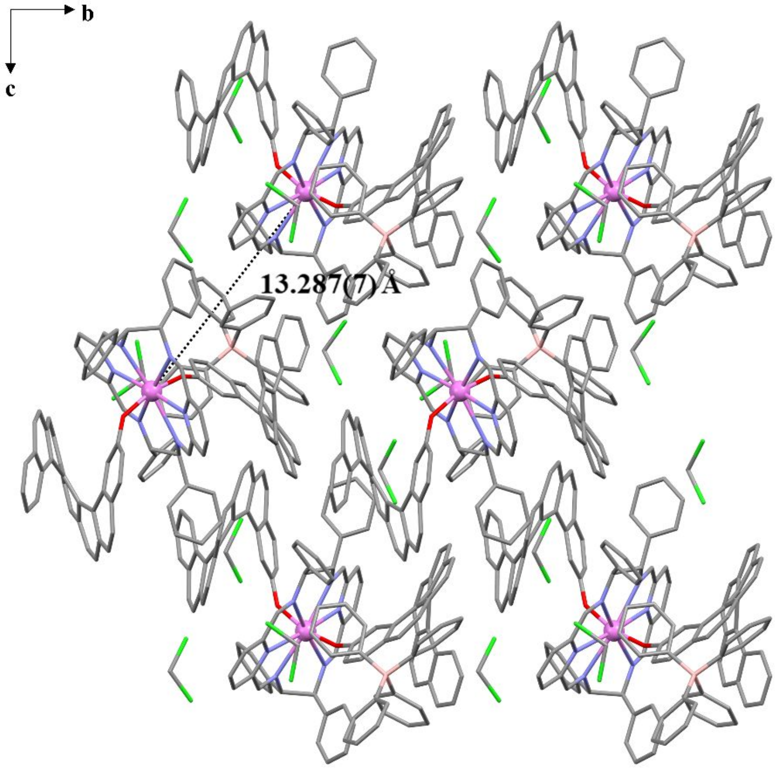

Figure S30. The packing diagram for **2** shown along the crystallographic *a* axis gives the shortest intermolecular Dy···Dy distance of 13.287(7) Å.

3. Magnetic measurements

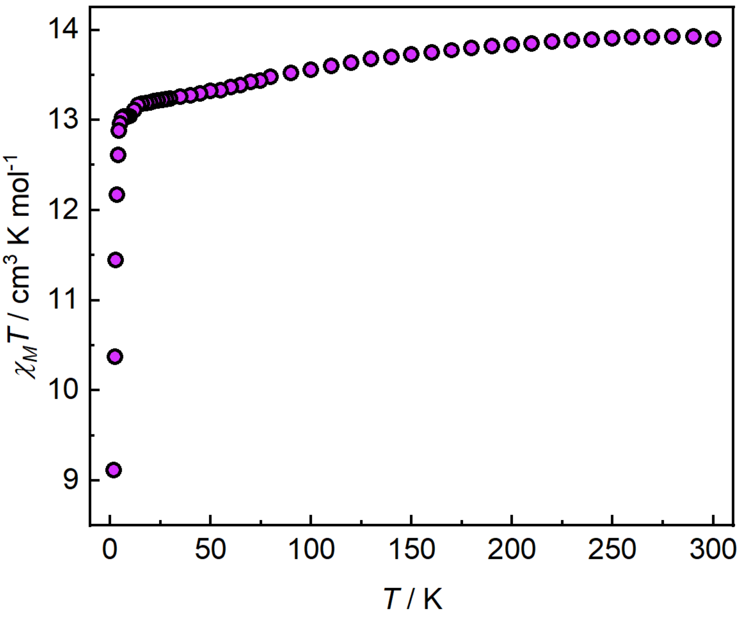

Figure S31. Plot of $\chi_M T$ versus temperature for **1** in an applied magnetic field of 1 kOe.

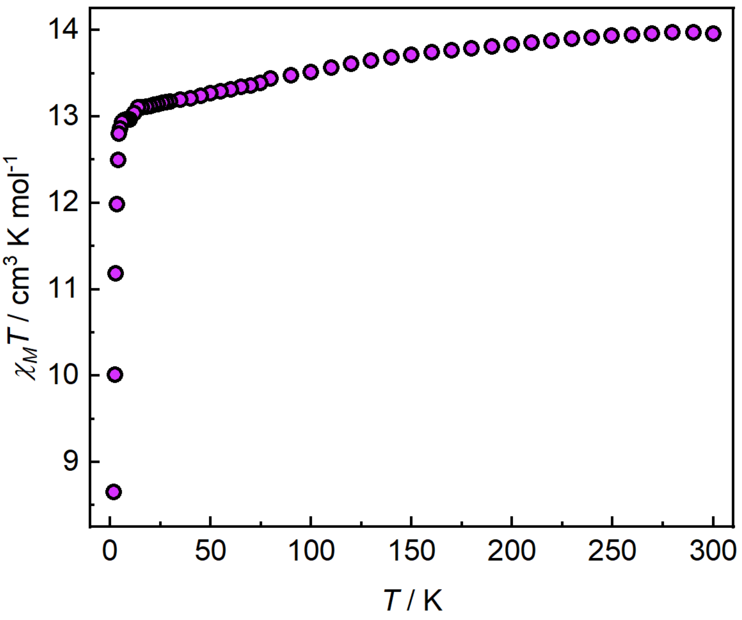

Figure S32. Plot of $\chi_M T$ versus temperature for **2** in an applied magnetic field of 1 kOe.

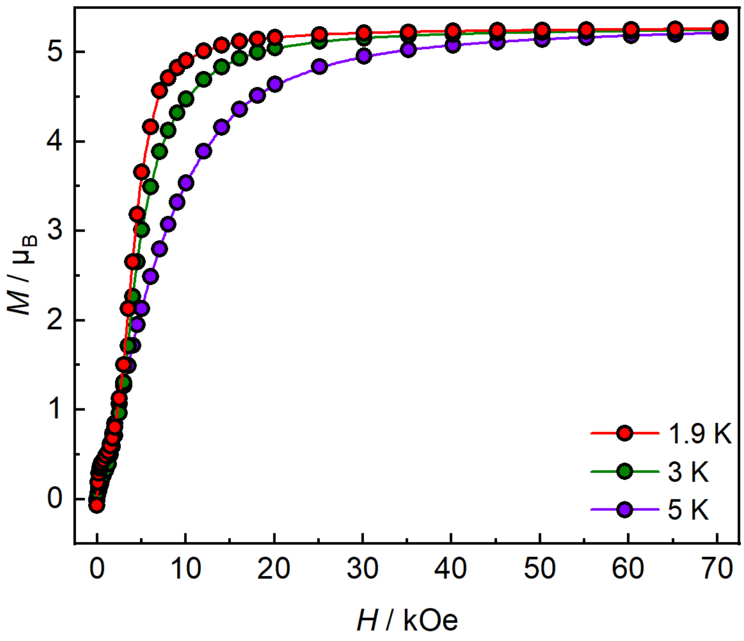

Figure S33. Field dependence of the magnetization at 1.9 K, 3 K and 5 K for **1**. $M = 5.26\ \mu_B$ at 1.9 K and 70 kOe.

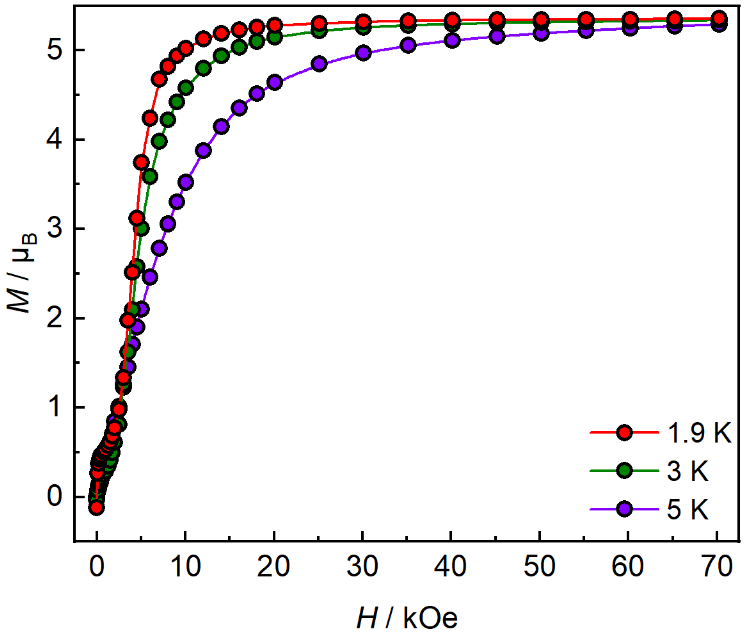

Figure S34. Field dependence of the magnetization at 1.9 K, 3 K and 5 K for **2**. $M = 5.36\ \mu_B$ at 1.9 K and 70 kOe.

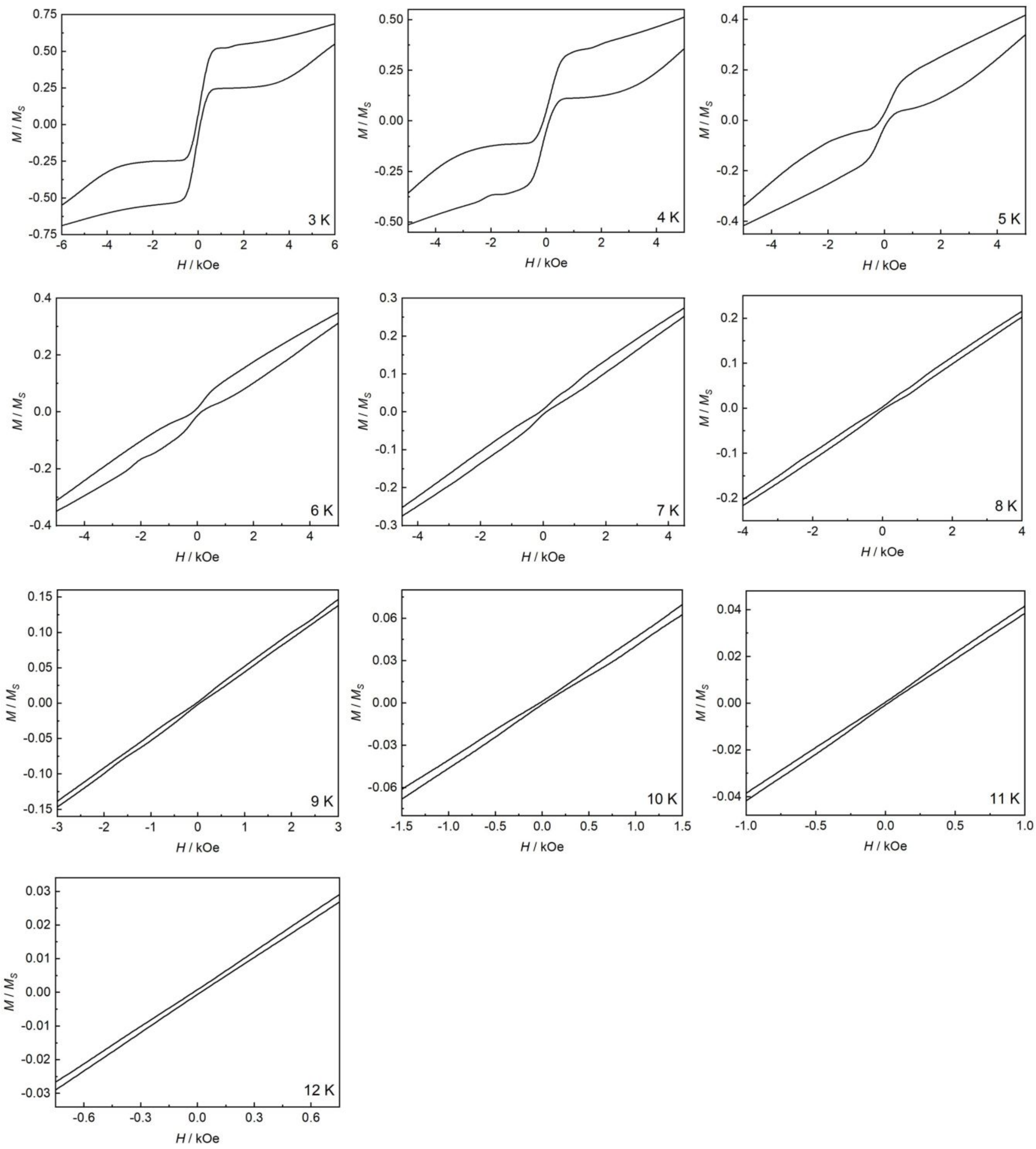

Figure S35. M(H) hysteresis loops for **1**. The data were collected continuously at different temperatures (from 3 to 12 K) using a sweep rate of 200 Oe/s.

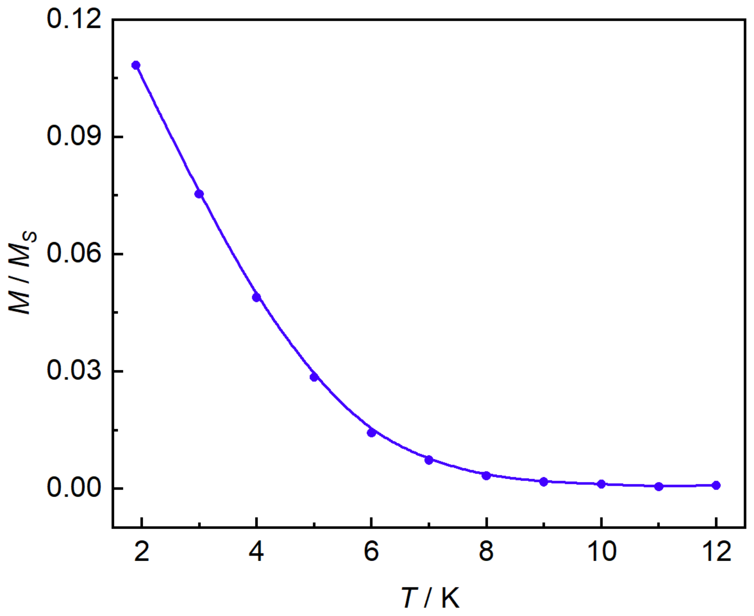

Figure S36. Plot of remnant magnetization vs temperature for **1**. Solid lines are guides for the eye.

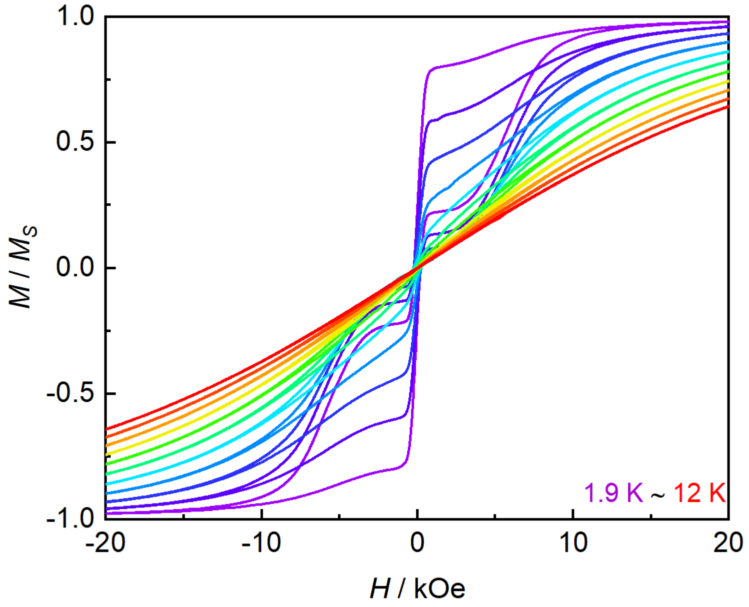

Figure S37. M(H) hysteresis loops for **2**. The data were collected continuously from 1.9 to 12 K using a sweep rate of 200 Oe/s.

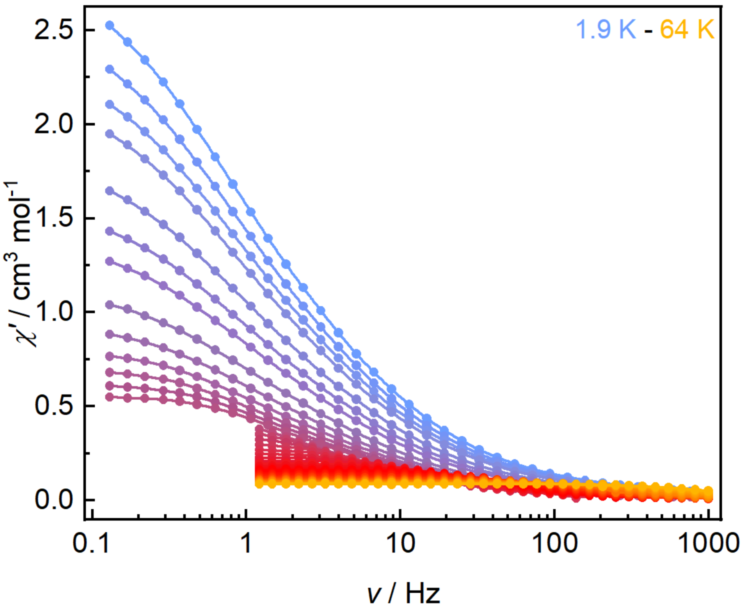

Figure S38. Frequency dependence of in-phase susceptibility (χ') for **1** in zero dc field.

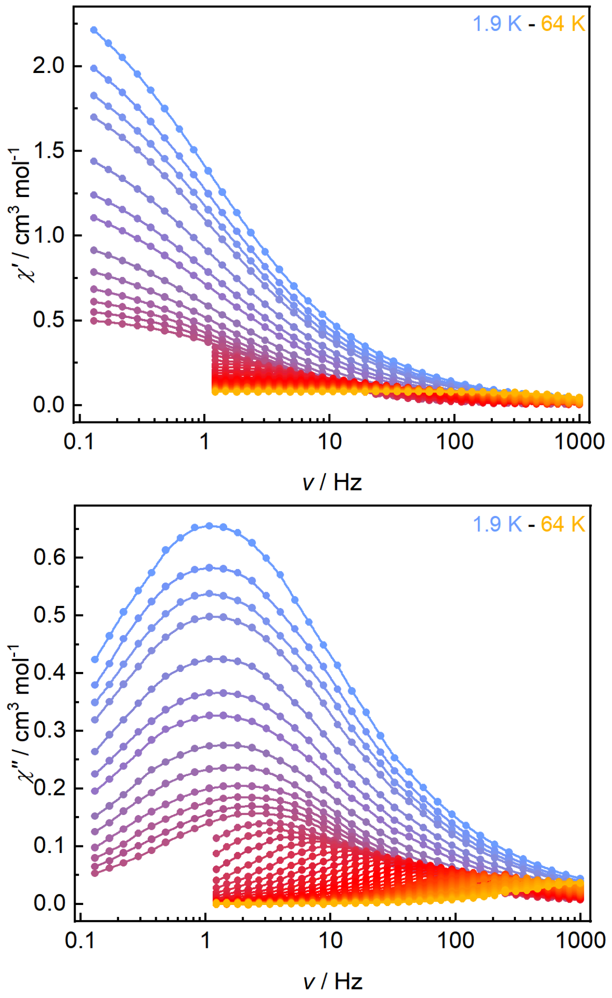

Figure S39. Frequency dependence of in-phase (χ') (top) and out-of-phase (χ'') susceptibility (bottom) for **2** in zero dc field.

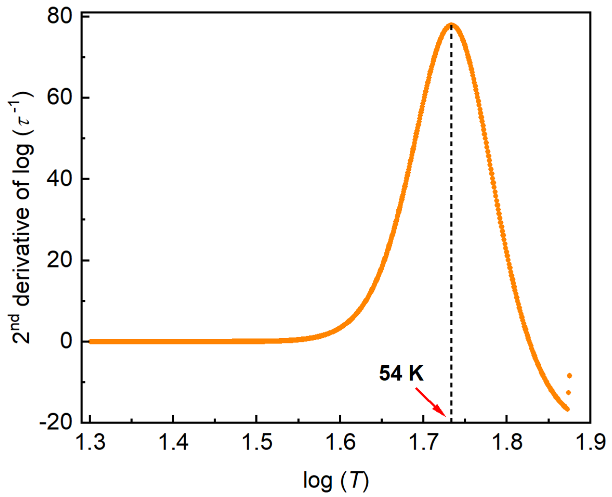

Figure S40. The second derivative of log(τ^{-1}) vs. log(T) plot for **1**. The maximum indicates the temperature at which the Raman relaxation mechanism becomes dominant over the Orbach process.

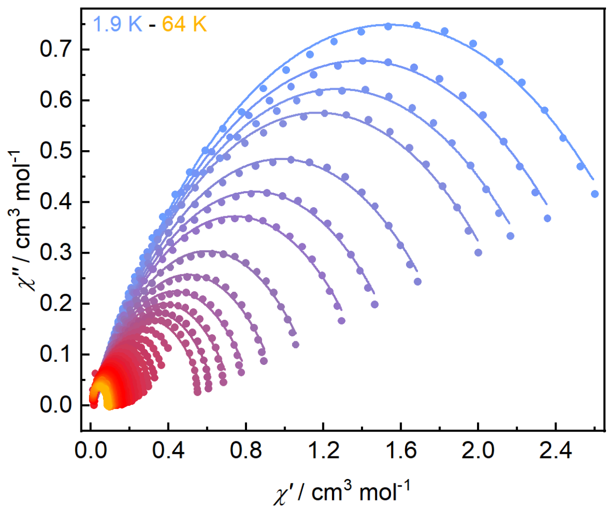

Figure S41. Cole-Cole plots for **1**. The solid lines are obtained by fitting experimental data to generalized Debye model.

Table S6. The relaxation fitting parameters for **1** from fitting ac data at varying temperatures.

T (K)	χ_s	χ_T	τ	α
1.9	0.175058E-14	0.295300E+01	0.138195E+00	0.414110E+00
2.1	0.131147E-14	0.267059E+01	0.134873E+00	0.413928E+00
2.3	0.189931E-14	0.245980E+01	0.133104E+00	0.413629E+00
2.5	0.259738E-14	0.227502E+01	0.130511E+00	0.412835E+00
3.0	0.287113E-14	0.191840E+01	0.125196E+00	0.412421E+00
3.5	0.260258E-14	0.166192E+01	0.119889E+00	0.410753E+00
4.0	0.328844E-14	0.145977E+01	0.112223E+00	0.407292E+00
5.0	0.440387E-14	0.118337E+01	0.101252E+00	0.404111E+00
6.0	0.652849E-14	0.100110E+01	0.923749E-01	0.402611E+00
7.0	0.776669E-14	0.876738E+00	0.859652E-01	0.403889E+00
8.0	0.143001E-13	0.786490E+00	0.810804E-01	0.404365E+00
9.0	0.111849E-13	0.715792E+00	0.753861E-01	0.402794E+00
10.0	0.525673E-14	0.643559E+00	0.661283E-01	0.396340E+00
12.0	0.253003E-14	0.547855E+00	0.509298E-01	0.375339E+00
14.0	0.561150E-14	0.454962E+00	0.348204E-01	0.338217E+00
16.0	0.109314E-13	0.386210E+00	0.245862E-01	0.301061E+00
18.0	0.201543E-13	0.335136E+00	0.178837E-01	0.266139E+00
20.0	0.308316E-13	0.294298E+00	0.131383E-01	0.235148E+00
22.0	0.438013E-13	0.263968E+00	0.102216E-01	0.208135E+00
24.0	0.672546E-13	0.239404E+00	0.794486E-02	0.184501E+00
26.0	0.110544E-12	0.218919E+00	0.631165E-02	0.164802E+00
28.0	0.156271E-12	0.201872E+00	0.511720E-02	0.143991E+00
30.0	0.270072E-12	0.188136E+00	0.415614E-02	0.137206E+00
32.0	0.452698E-12	0.175815E+00	0.341203E-02	0.124993E+00
34.0	0.653322E-12	0.164583E+00	0.287533E-02	0.113479E+00
36.0	0.937944E-12	0.155105E+00	0.238811E-02	0.105811E+00
38.0	0.134543E-11	0.146388E+00	0.201330E-02	0.979416E-01
40.0	0.217797E-11	0.139439E+00	0.173250E-02	0.100041E+00
42.0	0.274983E-11	0.133247E+00	0.148603E-02	0.938345E-01
44.0	0.411599E-11	0.126570E+00	0.127957E-02	0.877323E-01
45.0	0.576251E-11	0.123863E+00	0.118155E-02	0.856623E-01
46.0	0.858337E-11	0.120959E+00	0.109536E-02	0.865232E-01
47.0	0.112488E-10	0.118620E+00	0.101876E-02	0.850332E-01
48.0	0.149141E-10	0.116380E+00	0.943365E-03	0.881131E-01
49.0	0.211196E-10	0.113632E+00	0.873929E-03	0.825071E-01
50.0	0.286071E-10	0.111415E+00	0.805968E-03	0.826507E-01
51.0	0.407277E-10	0.109097E+00	0.744711E-03	0.818512E-01
52.0	0.521426E-10	0.106835E+00	0.678160E-03	0.777839E-01
53.0	0.697340E-10	0.105229E+00	0.629130E-03	0.799953E-01
54.0	0.103020E-09	0.103201E+00	0.560156E-03	0.741637E-01
55.0	0.130300E-09	0.101460E+00	0.526067E-03	0.103302E+00
56.0	0.217179E-09	0.992164E-01	0.446121E-03	0.687021E-01

57.0	0.354548E-09	0.976167E-01	0.399306E-03	0.778110E-01
58.0	0.524445E-09	0.960846E-01	0.350264E-03	0.764376E-01
59.0	0.792147E-09	0.942484E-01	0.304287E-03	0.773807E-01
60.0	0.120979E-08	0.929277E-01	0.260859E-03	0.829306E-01
61.0	0.176158E-08	0.910752E-01	0.220856E-03	0.784415E-01
62.0	0.264780E-08	0.897268E-01	0.186741E-03	0.841304E-01
63.0	0.373914E-08	0.883044E-01	0.155783E-03	0.865936E-01
64.0	0.485538E-08	0.870476E-01	0.133248E-03	0.984395E-01

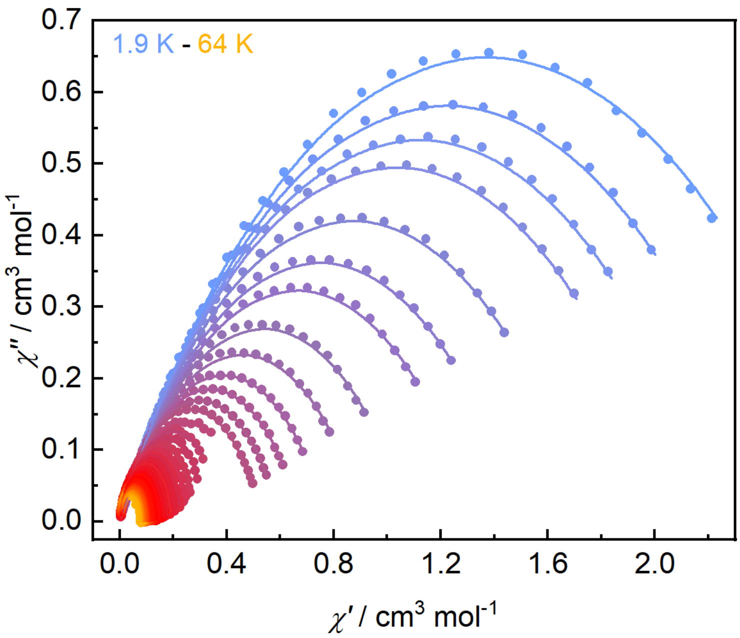

Figure S422. Cole-Cole plots for **2**. The solid lines are obtained by fitting experimental data to generalized Debye model.

Table S7. The relaxation fitting parameters for **2** from fitting ac data at varying temperatures.

T (K)	χ_s	χ_T	τ	α
1.9	0.477190E-03	0.271345E+01	0.140312E+00	0.429745E+00
2.1	0.313580E-15	0.244152E+01	0.139589E+00	0.431059E+00
2.3	0.454687E-15	0.223747E+01	0.136716E+00	0.429978E+00
2.5	0.622184E-15	0.206897E+01	0.133735E+00	0.427599E+00
3.0	0.852504E-15	0.174425E+01	0.131889E+00	0.424151E+00
3.5	0.116138E-14	0.149754E+01	0.128490E+00	0.423065E+00
4.0	0.160241E-14	0.132927E+01	0.126132E+00	0.420460E+00
5.0	0.211305E-14	0.107973E+01	0.116291E+00	0.406909E+00
6.0	0.300060E-14	0.915001E+00	0.108102E+00	0.396367E+00
7.0	0.423415E-14	0.777383E+00	0.932798E-01	0.380335E+00
8.0	0.592556E-14	0.677905E+00	0.807151E-01	0.360162E+00
9.0	0.126881E-13	0.599695E+00	0.694954E-01	0.342865E+00
10.0	0.197869E-13	0.535825E+00	0.598876E-01	0.324332E+00
12.0	0.475543E-03	0.480990E+00	0.540933E-01	0.325947E+00
14.0	0.271879E-02	0.393365E+00	0.360586E-01	0.271298E+00
16.0	0.413507E-02	0.335762E+00	0.256535E-01	0.224716E+00
18.0	0.469175E-02	0.289499E+00	0.182595E-01	0.182455E+00
20.0	0.490574E-02	0.258471E+00	0.137071E-01	0.151201E+00
22.0	0.461435E-02	0.233098E+00	0.104643E-01	0.131655E+00
24.0	0.432831E-02	0.211689E+00	0.810077E-02	0.112485E+00
26.0	0.386558E-02	0.195097E+00	0.635788E-02	0.100777E+00
28.0	0.384075E-02	0.180665E+00	0.510340E-02	0.864568E-01
30.0	0.367816E-02	0.167897E+00	0.413228E-02	0.747426E-01
32.0	0.404438E-02	0.157459E+00	0.344071E-02	0.654489E-01
34.0	0.327390E-02	0.148174E+00	0.285697E-02	0.597482E-01
36.0	0.319129E-02	0.139445E+00	0.239768E-02	0.494991E-01
38.0	0.330481E-02	0.132263E+00	0.204830E-02	0.428061E-01
40.0	0.307137E-02	0.125526E+00	0.175081E-02	0.364339E-01
42.0	0.301587E-02	0.119431E+00	0.150538E-02	0.296400E-01
44.0	0.284054E-02	0.113969E+00	0.130527E-02	0.275013E-01
45.0	0.282484E-02	0.111254E+00	0.121228E-02	0.242804E-01
46.0	0.285532E-02	0.108745E+00	0.112647E-02	0.214511E-01
47.0	0.312851E-02	0.106114E+00	0.105123E-02	0.153435E-01
48.0	0.186810E-02	0.104390E+00	0.940990E-03	0.269800E-01
49.0	0.272244E-02	0.102287E+00	0.905600E-03	0.174578E-01
50.0	0.253970E-02	0.100618E+00	0.839602E-03	0.216099E-01
51.0	0.244964E-02	0.987226E-01	0.774772E-03	0.211253E-01
52.0	0.269186E-02	0.969739E-01	0.715425E-03	0.193263E-01
53.0	0.576112E-02	0.948522E-01	0.690723E-03	0.341118E-01
54.0	0.293098E-02	0.930380E-01	0.592079E-03	0.101482E-01
55.0	0.249494E-02	0.916179E-01	0.531923E-03	0.196529E-01
56.0	0.281018E-02	0.897674E-01	0.477643E-03	0.138123E-01

57.0	0.269688E-02	0.881549E-01	0.426212E-03	0.140144E-01
58.0	0.289421E-02	0.867847E-01	0.380061E-03	0.752476E-02
59.0	0.282508E-02	0.856524E-01	0.327520E-03	0.158989E-01
60.0	0.283443E-02	0.842330E-01	0.286336E-03	0.193438E-01
61.0	0.211108E-02	0.829736E-01	0.241849E-03	0.281294E-01
62.0	0.263067E-02	0.815174E-01	0.206531E-03	0.259769E-01
63.0	0.293880E-02	0.802108E-01	0.174634E-03	0.285066E-01
64.0	0.866981E-15	0.791073E-01	0.141161E-03	0.548804E-01

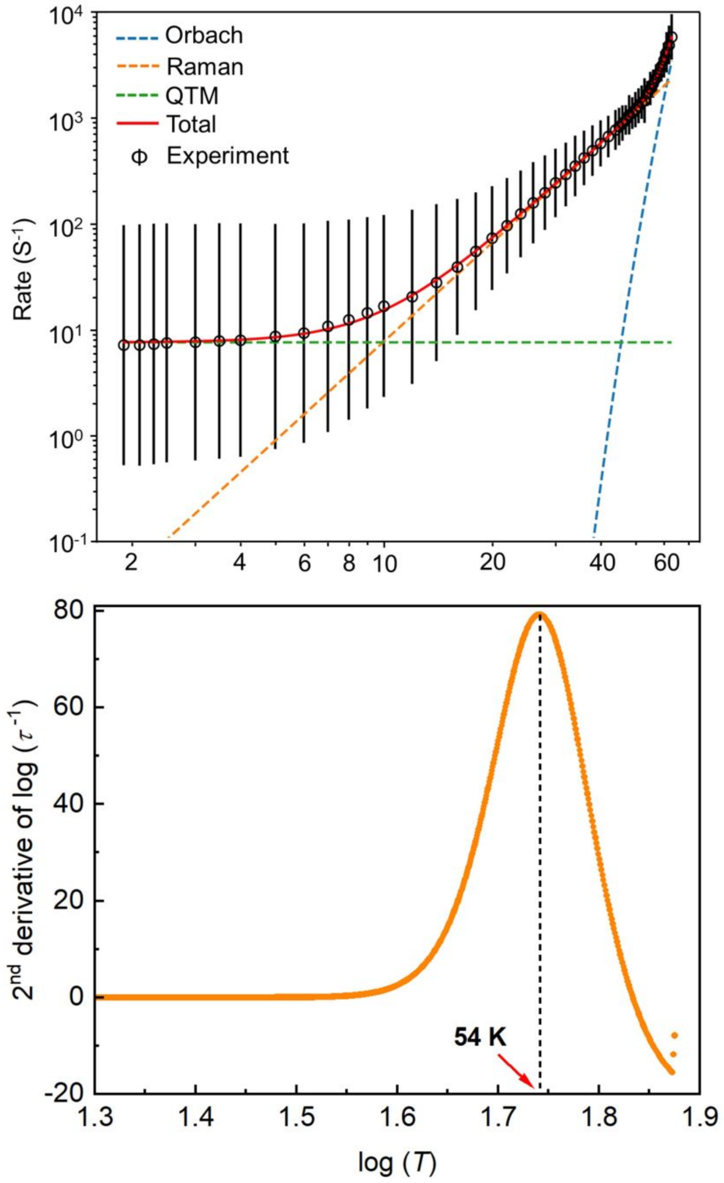

Figure S43. (Top) Temperature dependence of the magnetization reversal rate for **2**. Solid black lines indicate error bars from the distributions of relaxation times. The fitting equation is $\tau^{-1} = CT^{n} + \tau_0^{-1}\exp(-U_{eff}/k_BT) + \tau_{QTM}^{-1}$, giving U_{eff}/k_B = 615(12) cm^{-1}, τ_0 = 1.4(4)×10^{-10} s, C = 1.2(2) ×10^{-2} s^{-1} K^{-n}, n = 2.90(5), τ_{QTM} = 0.14(1) s, R^2=0.99823; (Bottom) The second derivative of log(τ^{-1}) vs. log(T) plot for **2**. The maximum indicates the temperature at which the Raman relaxation mechanism becomes dominant over the Orbach process.

Table S8. Parameters obtained from the fitting of τ^{-1} vs. T plots and theoretical calculations.

Parameters	**1** (Fitting)	**2** (Fitting)	**1** (calculating)	**2** (calculating)
C (s^{-1} K^{-n})	1.2(2)×10^{-2}	1.2(2)×10^{-2}	0.45×10^{-2}	1.06×10^{-2}
n	2.90(6)	2.90(5)	3.24	3.07
τ_0 (s)	1.2(4)×10^{-10}	1.4(4)×10^{-10}	4.3×10^{-10}	4.3×10^{-10}
U_{eff} (cm^{-1})	618(14)	615(12)	638	638

4. Theoretical calculations

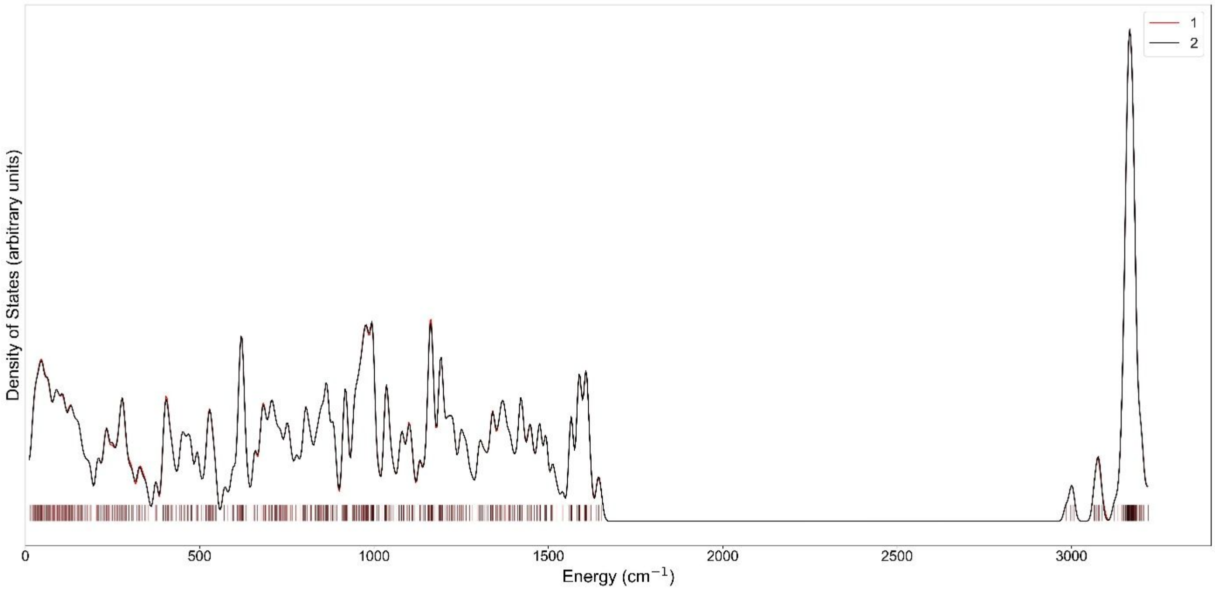

Figure S44. DFT phonons density of states for **1**(red) and **2**(black). Small vertical lines correspond to the computed phonon energies at Γ.

Table S9. Selected DFT bond distances (Å) for **1** and **2.**

	**1**	**2**
Dy-O1	2.128	2.13
Dy-O2	2.155	2.153
Dy-N1	2.646	2.637
Dy-N2	2.729	2.737
Dy-N3	2.637	2.625
Dy-N4	2.636	2.647
Dy-N5	2.627	2.638
Dy-N6	2.732	2.729

Table S10. CASSCF energies and *g*-factors of the first 8 Kramers doublets for compound **1**.

KD	Energy/cm^{-1}	g-factors		
KD1	0.0	0.000705	0.000794	19.964797
KD2	445.5	0.112383	0.119680	16.996149
KD3	791.0	0.038381	0.268956	13.802424
KD4	988.1	2.371549	3.211204	7.814571
KD5	1041.0	0.915845	7.405413	12.251520
KD6	1074.3	0.046052	5.211090	12.656645
KD7	1145.8	2.009010	2.735823	8.542770
KD8	1226.3	1.142529	4.558603	15.070819

Table S11. CASSCF energies and *g*-factors of the first 8 Kramers doublets for compound **2**.

KD	Energy/cm^{-1}	g-factors		
KD1	0.0	0.000772	0.000850	19.964483
KD2	445.3	0.112141	0.119789	16.995789
KD3	790.0	0.016986	0.244209	13.805607
KD4	986.3	2.771502	3.455273	7.944244
KD5	1039.7	0.824792	7.399499	12.020132
KD6	1074.9	0.123990	5.141658	12.570895
KD7	1144.3	1.925537	2.664154	8.485828
KD8	1222.8	1.161269	4.793793	14.862610

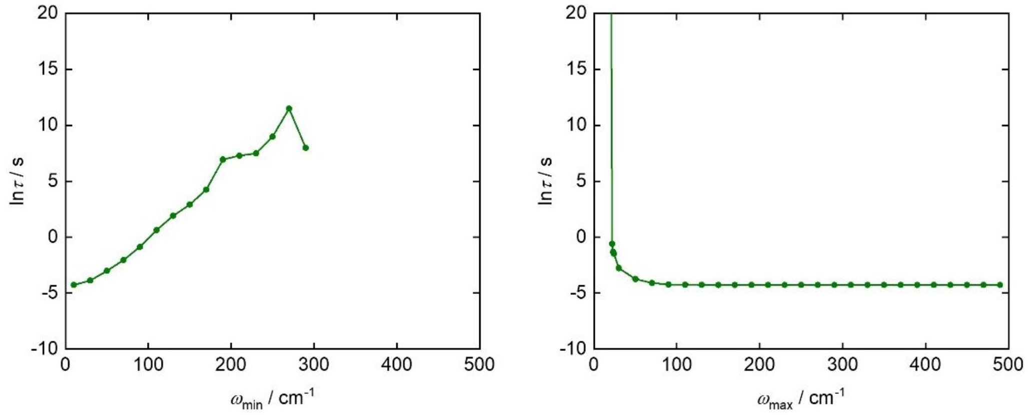

Figure S45. Raman contribution for **1** to τ as a function of low-energy cutoff ω_{min} (left) and as a function of high-energy cutoff ω_{max} (right). Temperature is fixed at 20 K.

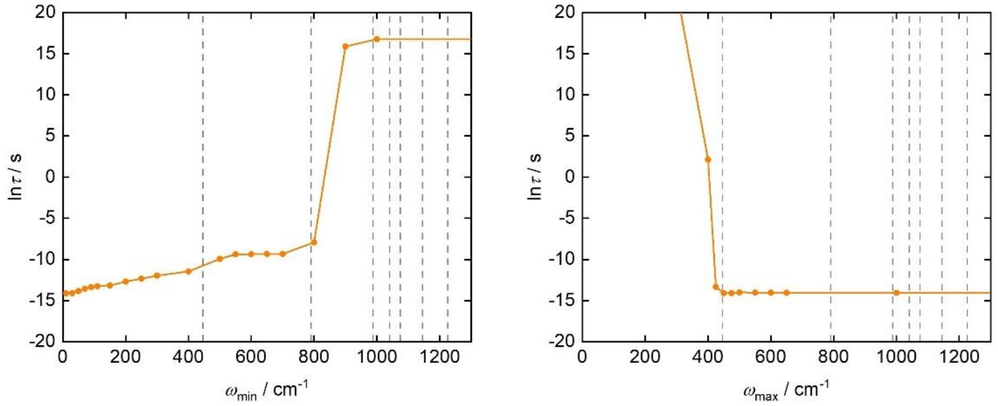

Figure S46. Orbach contribution for **1** to τ as a function of low-energy cutoff ω_{min} (left) and as a function of high-energy cutoff ω_{max} (right). Temperature is fixed at 100 K.

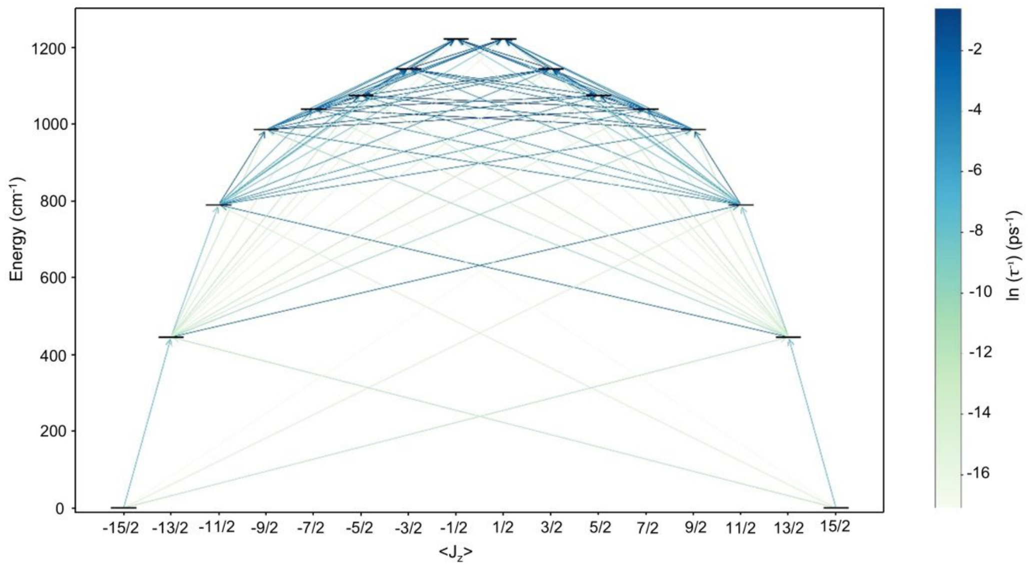

Figure S47. Computed Orbach transition rates, τ^{-1}, between energy states for **1** at 100 K. Larger values of τ^{-1} indicate more probable transitions.

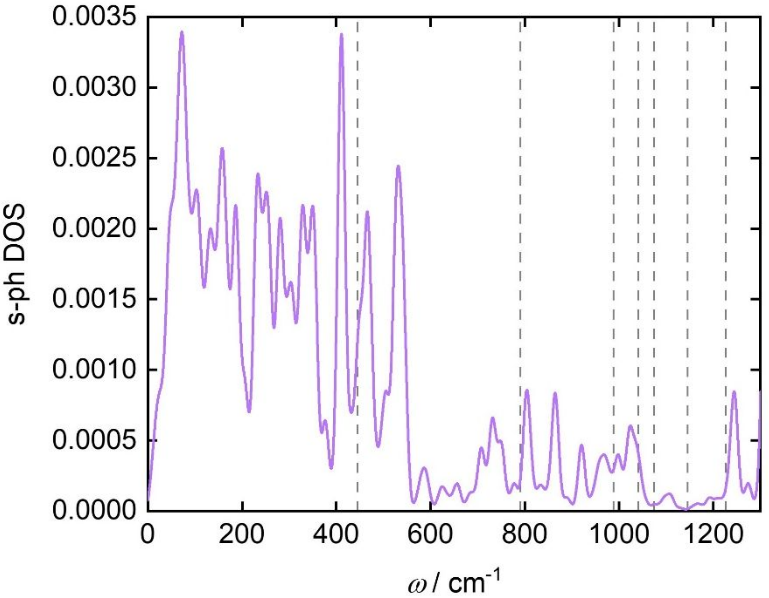

Figure S48. Spin-phonon coupling density of states $D(\omega)$ of **1**. Kramers doublet energy levels are reported as dashed lines.

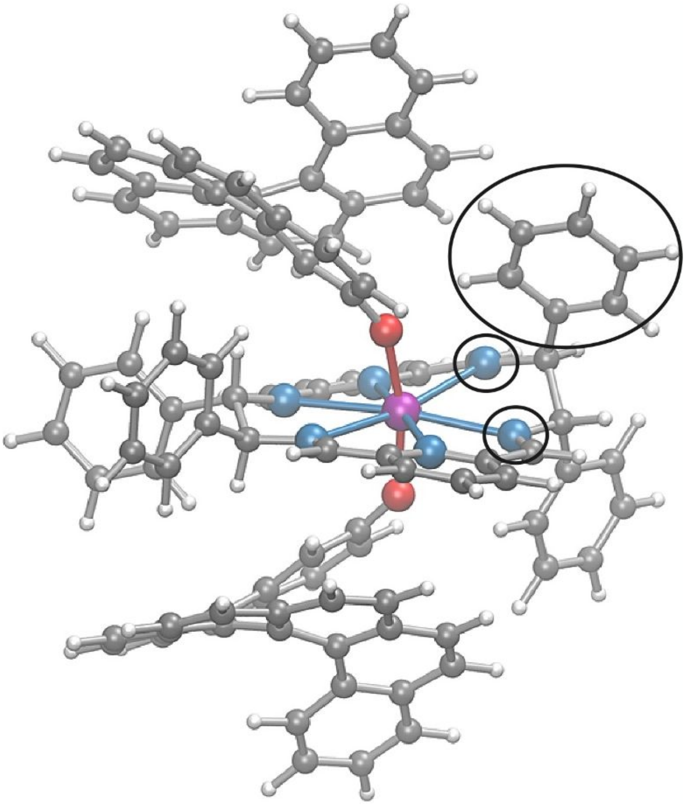

Figure S49. The groups of atoms primarily involved in the vibrations of modes 301, 302, 303, and 304 are circled in the molecular geometry of **2**.

5. References

1. Matsushima, T.; Kobayashi, S.; Watanabe, S., Air-Driven Potassium Iodide-Mediated Oxidative Photocyclization of Stilbene Derivatives. *J. Org. Chem.* **2016,** *81*, 7799-7806.
2. Jakubec, M. B., T.; Jakubík, P.; Sýkora, J.; Žádný, J.; Církva, V.; Storch, J., 2-Bromo[6]helicene as a Key Intermediate for [6]Helicene Functionalization. *J. Org. Chem.* **2018,** *83*, 3607-3616.
3. Boudreaux, E. A.; Mulay, L. N., Theory and Applications of Molecular Paramagnetism. John Wiley & Sons: New York, 1976.
4. Zhao, C.; Wang, T.; Liu, X.; Zhu, Z.; Ying, X.; Li, X.-L.; Tang, J., Peroxido-bridged chiral double-decker dysprosium macrocycles. *Dalton Trans.* **2023,** *52*, 15456-15461.
5. Dolomanov, O. V.; Bourhis, L. J.; Gildea, R. J.; Howard, J. A. K.; Puschmann, H., OLEX2: a complete structure solution, refinement and analysis program. *J. Appl. Crystallogr.* **2009,** *42*, 339-341.
6. Sheldrick, G. M., Crystal structure refinement with SHELXL. *Acta Crystallogr. C Struct. Chem.* **2015,** *71*, 3-8.
7. Sheldrick, G. M., SHELXT - integrated space-group and crystal-structure determination. *Acta Crystallogr. A Found. Adv.* **2015,** *71*, 3-8.
8. Pinsky, M.; Avnir, D., Continuous Symmetry Measures. 5. The Classical Polyhedra. *Inorg. Chem.* **1998,** *37*, 5575-5582.
9. Casanova, D.; Cirera, J.; Llunell, M.; Alemany, P.; Avnir, D.; Alvarez, S., Minimal Distortion Pathways in Polyhedral Rearrangements. *J. Am. Chem. Soc.* **2004,** *126*, 1755-1763.
